# Cognitive Phenotyping and Interpretation of Alzheimer Blood Biomarkers

**DOI:** 10.1001/jamaneurol.2025.0142

**Published:** 2025-04-04

**Authors:** Vincent Bouteloup, Nicolas Villain, Jean Sebastien Vidal, Fernando Gonzalez-Ortiz, Idil Yuksekel, Cristiano Santos, Susanna Schraen-Maschken, Isabelle Pellegrin, Sylvain Lehmann, Kaj Blennow, Geneviève Chêne, Olivier Hanon, Carole Dufouil, Vincent Planche

**Affiliations:** 1 Bordeaux Population Health, University of Bordeaux, Inserm, UMR1219, Bordeaux, France; 2 CIC 1401 EC, Pôle Santé Publique, CHU de Bordeaux, Bordeaux, France; 3 Sorbonne Université, INSERM U1127, CNRS 7225, Institut du Cerveau–ICM, Paris, France; 4 Department of Neurology, Institute of Memory and Alzheimer’s Disease, AP-HP Sorbonne Université, Pitié-Salpêtrière Hospital, Paris, France; 5 Memory Resource and Research Centre of Paris-Broca-Ile de France, APHP, Hôpitaux Universitaires Paris Centre, Hospital Broca, Paris, France; 6 Université Paris Cité, EA 4468, Paris, France; 7 Inst. of Neuroscience and Physiology, University of Gothenburg, Mölndal, Sweden; 8 Clinical Neurochemistry Lab, Sahlgrenska University Hospital, Mölndal, Sweden; 9 UMR-S1172 Lille Neuroscience & Cognition, University of Lille, Inserm, CHU Lille, Lille, France; 10 Laboratory of Immunology and Immunogenetics, Resources Biological Center, CHU Bordeaux, Bordeaux, France; 11 Univ. Bordeaux, CNRS, ImmunoConcEpT, UMR 5164, Bordeaux, France; 12 LBPC-PPC, Univ Montpellier, INM INSERM, CHU Montpellier, Montpellier, France; 13 Institut des Maladies Neurodégénératives, Univ. Bordeaux, CNRS, UMR 5293, Bordeaux, France; 14 Pôle de Neurosciences Cliniques, Centre Mémoire de Ressources et de Recherche, CHU de Bordeaux, Bordeaux, France

## Abstract

**Importance:**

Blood phosphorylated tau 217 (p-tau217) showed good performance in predicting brain amyloidosis. However, the importance of detailed cognitive phenotyping in patients without dementia when interpreting p-tau217 results remains unclear.

**Objective:**

To assess whether accuracy, negative predictive value (NPV), and positive predictive value (PPV) in predicting brain amyloidosis using p-tau217 varies across clinical presentations in patients without dementia.

**Design, Setting, and Participants:**

The study design included 2 observational, prospective cohort studies: The Cohort of Outpatients From French Research Memory Centers in Order to Improve Knowledge on Alzheimer’s Disease and Related Disorders (MEMENTO), with enrollment from 2011 to 2014 and 5 years of follow-up, and the Biomarker of Amyloid Peptide and Alzheimer’s Disease Risk (BALTAZAR) cohort study, with enrollment from 2010 to 2015 and 3 years of follow-up. Both are multicenter cohorts conducted in French memory clinics. Participants without dementia were included for analysis if they had baseline blood p-tau217 measurement and a known amyloid status through cerebrospinal fluid amyloid β (Aβ)–42/Aβ-40 ratio or positron emission tomography. They presented with either subjective cognitive impairment (SCI), mild cognitive impairment (MCI) with a common Alzheimer disease (AD) phenotype (cAD-MCI: amnestic syndrome of hippocampal type, posterior cortical atrophy, or logopenic primary progressive aphasia), or MCI with uncommon AD or other phenotypes (uAD-MCI). Data were analyzed from May to September 2024.

**Exposures:**

Blood p-tau217 concentrations.

**Main Outcomes and Measures:**

Brain amyloidosis probabilities were derived from p-tau217 logistic regressions including age, gender, and *APOE* genotype. Published and internally developed cut points with 90% sensitivity and specificity were used.

**Results:**

A total of 776 participants from the MEMENTO cohort (N = 2323 participants) and 193 participants from the BALTAZAR cohort (N = 1040) were included in this analysis. In the MEMENTO cohort (median [IQR] age, 71 [65-76] years; 444 female [57%]), brain amyloidosis prevalence was 16.5% (20 of 121) in SCI, 45.9% (78 of 170) in cAD-MCI, and 24.5% (119 of 485) in uAD-MCI. Area under the receiver operating characteristic curve for predicting brain amyloidosis with p-tau217 models was 0.78 (95% CI, 0.66-0.89), 0.91 (95% CI, 0.86-0.95), and 0.87 (95% CI, 0.84-0.91) in the SCI, cAD-MCI, and uAD-MCI subgroups, respectively. External cut points resulted in a PPV of 60.0%, 90.0%, and 74.5% in the SCI, cAD-MCI, and uAD-MCI subgroups, respectively. NPV ranged from 84.2% to 90.2%. With internally developed cut points, PPVs were 52.6%, 84.0%, and 72.3% in the SCI, cAD-MCI, and uAD-MCI subgroups, respectively. NPVs were high (91.7%-94.6%) in all subgroups. Rates of incident dementia strongly increased with the probability of brain amyloidosis in the cAD-MCI subgroup. Replicated analyses in the BALTAZAR cohort provided similar results.

**Conclusions and Relevance:**

Results from 2 clinical cohorts suggest that amyloid prevalence varied across cognitive phenotypes and was associated with the diagnostic performance of blood p-tau217 models to determine brain amyloidosis. Comprehensive cognitive phenotyping beyond the basic characterization of SCI, MCI, or dementia should accompany the use of blood biomarkers in clinical practice to avoid misdiagnosis due to false positives.

## Key Points

**Question:**

Is the clinical phenotype of patients without dementia associated with blood phosphorylated tau 217 (p-tau 217) interpretation?

**Findings:**

In this study including 969 individuals from 2 clinic-based cohorts, participants without dementia were classified according to their neuropsychological symptoms, distinguishing between cognitive complaints, common Alzheimer disease (AD) phenotypes, and uncommon AD phenotypes. Significant differences were observed in the accuracy of p-tau217 to predict brain amyloidosis and in the positive predictive values, using both published and internally developed diagnostic cut points.

**Meanings:**

Results suggest that cognitive phenotyping was pivotal for sound interpretation of blood p-tau217 concentrations in memory clinics because it determines pretest probability of being amyloid positive.

## Teaser

This cohort study investigates if the clinical phenotype of patients without dementia is associated with blood phosphorylated tau 217 interpretation.

## Introduction

Current European recommendations for diagnosing Alzheimer disease (AD) and neurocognitive disorders in memory clinics advocate for a comprehensive neuropsychological evaluation to precede cerebrospinal fluid (CSF) or positron emission tomography (PET) examination, with their prescription contingent on the clinical manifestation.^1^ The ease of access and reduced cost of blood-based biomarkers prompts a reassessment of this approach, and an update of these guidelines is ongoing. Some authors advocate for the broad use of blood biomarkers, particularly phosphorylated tau 217 (p-tau217), in individuals with cognitive impairment consulting memory clinics to predict AD and inform clinical decisions.^2,3,4^ Nonetheless, challenges remain due to overlapping blood biomarker concentrations between amyloid-positive (A+) and amyloid-negative (A−) groups^5^ and the influence of comorbidities and risk factors.^6^ To address these challenges, a dual cut point strategy has been proposed,^4,7^ categorizing patients into high, low, and intermediate risks of brain amyloidosis. Only those with intermediate risk would require confirmatory testing with CSF or PET.

Two paradigms are internationally recognized regarding the definition of AD. The Alzheimer Association framework states that compelling evidence of brain amyloidosis using abnormal core 1 biomarkers is sufficient to establish an AD diagnosis and to inform clinical decision.^8^ In contrast, the International Working Group (IWG) framework^9,10^ requires the identification of specific cognitive phenotypes together with abnormal biomarkers for an AD diagnosis,^11^ cautioning against overattributing cognitive decline to AD, especially in cases with mild cognitive impairment (MCI). The IWG defines *common AD phenotypes* as amnestic variant of hippocampal type, posterior cortical atrophy, or logopenic variant of primary progressive aphasia; *uncommon AD phenotypes* as including behavioral or dysexecutive variant, corticobasal syndrome, semantic, or nonfluent variants of primary progressive aphasia; and *other phenotypes* as Lewy body dementia, Richardson syndrome, and others. These clinical phenotypes present different prevalence of AD neuropathological changes (ADNC). ADNC are observed in 50% to 95% of common AD phenotypes cases,^9,12,13,14^ in 5% to 30% of uncommon phenotypes cases,^9,15^ and are theoretically not observed in other phenotypes, except as copathology.^9,10^ Thus, cognitive subtyping of patients with MCI should theoretically impact A+ pretest probability and the predictive values of diagnostic tests for ADNC.^16^

This study assessed whether accuracy and predictive values in predicting amyloid status using blood p-tau217 varied across cognitive phenotypes, as defined by the IWG. In The Cohort of Outpatients From French Research Memory Centers in Order to Improve Knowledge on Alzheimer’s Disease and Related Disorders (MEMENTO),^17^ we tested previously published cut points to predict brain amyloidosis with blood p-tau217. We also developed internal cut points in MEMENTO subsequently tested in the Biomarker of Amyloid Peptide and Alzheimer’s Disease Risk (BALTAZAR) cohort.^18^ We finally assessed the clinical prognosis at 3 and 5 years based on the clinical phenotype and the amyloid-positive probability as determined by blood p-tau217 concentrations.

## Methods

### Study Population: Main Sample

From 2011 to 2014, the MEMENTO cohort consecutively enrolled 2323 participants without dementia from 26 French memory clinics.^17^ For inclusion, participants were required to present a cognitive complaint alongside a Clinical Dementia Rating score of 0.5 or less. Main exclusion criteria were a history of head trauma with persistent neurological deficits, stroke in the last 3 months or with persistent neurological deficits, brain tumor, epilepsy, schizophrenia, known variation in familial AD genes, and illiteracy. All participants provided written informed consent. The study protocol received approval from the ethics committee CPP Sud-Ouest et Outre-Mer III. Race and ethnicity data were not available in the MEMENTO and BALTAZAR cohorts. French law does not permit the collection of race or ethnicity information in research studies. This work complied with the Strengthening the Reporting of Observational Studies in Epidemiology (STROBE) reporting guidelines.

At inclusion and yearly over a 5-year period, participants underwent a comprehensive clinical, standardized and exhaustive neuropsychological assessment as previously described.^17^ Participants who exhibited a cognitive complaint without any objective impairment in any test of the baseline neuropsychological test battery (1.5 SD below the appropriate norm) were considered to have subjective cognitive impairment (SCI). Conversely, individuals presenting impairment in at least 1 test were classified as having MCI.^19^ All incident dementia cases underwent review by an expert panel, blinded to any genetic or biological biomarker information.

### AD Cognitive Phenotypes in Participants With MCI

Phenotypes of patients with MCI at baseline were further categorized according to the last IWG recommendations for the diagnosis of AD^9,10^: those with an amnestic syndrome of hippocampal type (inability to recall verbal information despite semantic cueing and encoding control, indicating the presence of a storage deficit), or posterior cortical atrophy or logopenic variant primary progressive aphasia were considered presenting a common AD phenotype (cAD-MCI). The amnestic syndrome of hippocampal type was defined by a sum of the 3 free and cued recalls of the Free and Cued Selective Reminding Test less than or equal to 42/48.^20^ The presence of posterior cortical atrophy or a logopenic variant primary progressive aphasia was reported by the local investigator based on international criteria.^21,22^ Individuals with MCI not presenting a common AD phenotype were categorized in the uncommon AD/other phenotypes group (uAD-MCI), which comprised behavioral and/or dysexecutive syndrome, corticobasal syndrome, possible or probable prodromal dementia with Lewy bodies,^23^ and other primary progressive aphasia.^9^

### Blood Biomarkers and *APOE* Genotype

Baseline blood samples were collected and centrally stored at −80 °C in the Genomic Analysis Laboratory-Biological Resource Centre (LAG-CRB) at the Pasteur Institute, Lille, France. Plasma p-tau217 was quantified using the University of Gothenburg (UGOT) p-tau217 assay,^24^ on a Quanterix HD-X analyzer at the Paris Brain Institute. Apolipoprotein E (*APOE*) genotypes were determined at LAG-CRB by KBiosciences.

### Detection of Amyloid Status

CSF collection or amyloid PET were optional examinations in the MEMENTO cohort. CSF was collected into polypropylene tubes following standardized protocols and centrally stored at −80 °C. CSF Amyloid-β 42 peptide (Aβ-42) and Aβ-40 were determined using Fujirebio INNOTEST kits. A predetermined CSF Aβ-42/Aβ-40 ratio less than 0.065 defined a pathological level of brain amyloid.^25^ PET imaging was proposed through 2 ancillary studies, Alzheimer’s Predictors in Subjective Memory Complainers (Insight-PreAD)^26^ and Longitudinal Study of Brain Amyloid Imaging in MEMENTO (MEMENTO-AMYGING).^27^ The radiotracer administered could be 18F-florbetapir or 18F-flutemetamol. We used predetermined cutoffs to define amyloid positivity (A+), as previously described.^5^

### Replication Sample

The BALTAZAR study is a French multicentric prospective clinical cohort.^18^ Among the 1040 individuals with dementia or MCI who were enrolled, the replication sample included individuals with MCI with a known amyloid status and a baseline blood p-tau217 measurement. A+ was defined by CSF Aβ-42/Aβ-40 ratio according to published norms.^28^ Blood p-tau217 concentrations were centrally determined using p217-ALZPath kits, on a Quanterix HD-X analyzer.^29^ Individuals were followed up for 3 years. Diagnoses of incident dementia were reviewed by an expert committee based on clinical and neuropsychological information.^30^ Definition of the cAD-MCI and uAD-MCI cognitive phenotypes followed the same definition as in the MEMENTO cohort.

### Statistical Analysis

#### Global Description

Descriptive data were presented using median and IQR or frequency and percentage. Group comparisons were done with Wilcoxon rank tests for quantitative variables and χ^2^ tests for categorical ones. Dementia onset was described through Kaplan-Meier survival curves.

#### Prediction of Amyloid Status

The probability of being A+ was estimated with regression logistic models including age, sex, *APOE* genotype, and blood p-tau217 concentration (log-transformed) as predictors. A model was fitted independently of the cognitive phenotype and separately in MEMENTO and BALTAZAR cohorts. Model performances were estimated with area under the curve (AUC).

#### External Cut Points Validation

First, we considered the cut points determined in Biomarkers For Identifying Neurodegenerative Disorders Early and Reliably (BioFINDER)-1 and BioFINDER-2 studies,^4^ defined for a given sensitivity and specificity at 90%. In these studies, probabilities less than 42%, 42% to 70%, and greater than 70% indicated individuals at low, intermediate, and high risk, respectively, of being A+. These cut points were applied in the MEMENTO cohort to estimate their predictive values across the different phenotype subgroups.

#### Development of Blood Biomarker Model Cut Points to Determine Amyloid Status

Second, we established in the MEMENTO cohort low, intermediate, and high probabilities of being A+ using a dual cut point strategy,^16^ independent of the cognitive phenotype, to reach prespecified sensitivity of 90% for the lowest one and specificity of 90% for the highest one. 95% CIs were estimated with 1000 bootstrap samples. These cut points were subsequently applied in the BALTAZAR independent replication sample. Three sensitivity analyses were performed: (1) using a definition of sensitivity = specificity = 95% cut points, (2) removing *APOE* genotype from the probability models, and (3) examining the predictive values according to the criterion standard used to determine amyloid status (CSF or PET).

#### Accuracy in Amyloid Status Detection

For both external and developed cut points, patients were categorized A− if their estimated probability fell below the lower cut point, A+ if their probability exceeded the higher cut point, and undetermined in between. Based on the cognitive phenotypes, we computed the positive predictive value (PPV), ie, the probability of being A+ using PET or CSF among individuals categorized positive by blood biomarkers, and the negative predictive value (NPV), ie, the probability of being A− among individuals categorized negative.

#### Dementia Incidence According to A+ Probability

Incidence rates of dementia per 100 person-years in cAD-MCI and uAD-MCI subgroups according to their A+ probabilities were estimated at 3 years (MEMENTO and BALTAZAR cohorts) and 5 years (MEMENTO cohort). Low, intermediate, and high probabilities of being A+ were determined using the internally developed cut points. Data were analyzed from May to September 2024 using R, version 4.3.3 (R Foundation for Statistical Computing). All *P* values were 2-sided, and a *P* value <.05 was considered statistically significant.

## Results

### Characteristics of the Study Samples

A total of 776 participants from the MEMENTO cohort (N = 2323 participants) and 193 participants from the BALTAZAR cohort (N = 1040) were included in this analysis. In the MEMENTO cohort (median [IQR] age, 71 [65-76] years; 444 female [57%]; 332 male [43%]), participants had known amyloid status and baseline blood p-tau217 quantification available. Overall, 121 patients presented with SCI, whereas 655 had MCI, 170 had the cAD-MCI phenotype (including 1 case of logopenic primary progressive aphasia, 1 case of posterior cortical atrophy, and 190 cases of amnestic syndrome of the hippocampal type), and 485 had the uAD-MCI phenotype (Table 1). A+ prevalence was 16.5% (20 of 121), 45.9% (78 of 170), and 24.5% (119 of 485) in the SCI, cAD-MCI, and uAD-MCI subgroups, respectively. In the BALTAZAR cohort (median [IQR] age, 78 [74-81] years; 117 female [61%]; 76 male [39%]), 119 participants had cAD-MCI (A+ frequency = 60.5%), and 74 individuals had uAD-MCI (A+ frequency = 43.2%).

**Table 1.** Baseline Characteristics According to Cognitive Phenotype in the Development and Replication Samples

Characteristic	Main: MEMENTO (n = 776)			Replication: BALTAZAR (n = 193)	
	SCI (n = 121)	MCI		MCI	
		With common AD (n = 170)	With uncommon AD/other (n = 485)	With common AD (n = 119)	With uncommon AD/other (n = 74)
Age, median (IQR), y	71 (65-75)	71 (66-76)	71 (65-76)	78 (75-82)	76 (73-80)
Sex, No. (%)					
Female	76 (62.8)	71 (41.8)	297 (61.2)	63 (52.9)	54 (73.0)
Male	45 (37.2)	99 (58.2)	188 (38.8)	56 (47.1)	20 (27.0)
High education level, No. (%)^a^	106 (72.6)	89 (46.4)	340 (61.3)	57 (47.9)	43 (58.1)
MMSE score, median (IQR)	29 (29-30)	27 (26-28)	29 (28-29)	26 (25-28)	28 (26-29)
FCSCRT total recall, median (IQR)	47 (46-48)	39 (33-41)	47 (45-48)	34 (25-39)	46 (44-47)
Blood p-tau217, median (IQR), pg/mL^b^	1.3 (1.0-2.0)	1.8 (1.1-3.0)	1.5 (1.0-2.0)	0.6 (0.3-0.8)	0.3 (0.2,0.7)
APOE eps4 allele, No. (%)					
Noncarriers	93 (76.9)	93 (54.7)	342 (70.5)	71 (59.7)	54 (73.0)
Heterozygous	24 (19.8)	61 (35.9)	132 (27.2)	39 (32.8)	17 (23.0)
Homozygous	4 (3.3)	16 (9.4)	11 (2.3)	9 (7.6)	3 (4.1)
Amyloid positivity, No. (%)^c^	20 (16.5)	78 (45.9)	119 (24.5)	72 (60.5)	32 (43.2)
Examination used to determine amyloid status, No. (%)					
CSF Aβ 42/40	44 (36.4)	107 (62.9)	175 (36.1)	119 (100)	74 (100)
PET florbetapir	54 (44.6)	38 (22.4)	224 (46.2)	NA	NA
PET flutemetamol	23 (19.0)	25 (14.7)	86 (17.7)	NA	NA
Amyloid quantification, median (IQR)					
CSF Aβ 42/40^d^	0.092 (0.065-0.117)	0.062 (0.042-0.103)	0.088 (0.062-0.111)	0.083 (0.055-0.148)	0.117 (0.084-0.159)
Florbetapir SUVR^e^	0.740 (0.686-0.832)	0.756 (0.676-1.232)	0.737 (0.690-0.842)	NA	NA
Flutemetamol SUVR^e^	0.663 (0.614-0.698)	0.638 (0.584-1.233)	0.648 (0.585-0.950)	NA	NA

Abbreviations: Aβ, amyloid beta; AD, Alzheimer disease; APOE, apolipoprotein E; BALTAZAR, Biomarker of Amyloid Peptide and Alzheimer’s Disease Risk study; CSF, cerebrospinal fluid; FCSCRT, free and cued selective reminding test; MCI, mild cognitive impairment; MEMENTO, The Cohort of Outpatients From French Research Memory Centers in Order to Improve Knowledge on Alzheimer’s Disease and Related Disorders study; MMSE, Mini Mental State Examination; NA, not applicable; p-tau217, phosphorylated tau 217; PET, positron emission tomography; SCI, subjective cognitive impairment; SUVR, standardized uptake value ratio.

^a^ Defined as French baccalaureate diploma (end of high school) or above.

^b^ MEMENTO: University of Gothenburg p-tau217 assay; BALTAZAR: p217-ALZPath assay.

^c^ Based on CSF Aβ 42/40 or PET imaging for MEMENTO, CSF Aβ 42/40 for BALTAZAR.

^d^ Threshold for amyloid positivity: 0.065 in MEMENTO, 0.060 in BALTAZAR.

^e^ Threshold for amyloid positivity: SUVR>0.88 for florbetapir, SUVR>1.063 for flutemetamol.

In the MEMENTO cohort, individuals presenting with a cAD-MCI phenotype were more likely to develop dementia during the 5-year follow-up period (eFigure in Supplement 1). A+ individuals with uAD-MCI had intermediate conversion rate, whereas A− individuals with uAD-MCI and A+ or A− individuals with SCI presented with the lowest dementia incidence rates. The same pattern was observed in the BALTAZAR cohort (eFigure in Supplement 1).

### Accuracy of Predicting Amyloid Status

In the MEMENTO cohort, the AUC to predict amyloid status with p-tau217 was 0.88 (95% CI, 0.85-0.91) overall. Discriminative performances were 0.78 (95% CI, 0.66-0.89), 0.91 (95% CI, 0.86-0.95), and 0.87 (95% CI, 0.84-0.91) in the SCI, cAD-MCI, and uAD-MCI subgroups, respectively (eTable 1 in Supplement 1). Accuracies were slightly higher in the BALTAZAR cohort.

### External Cut Point Validation

**Figure 1.**
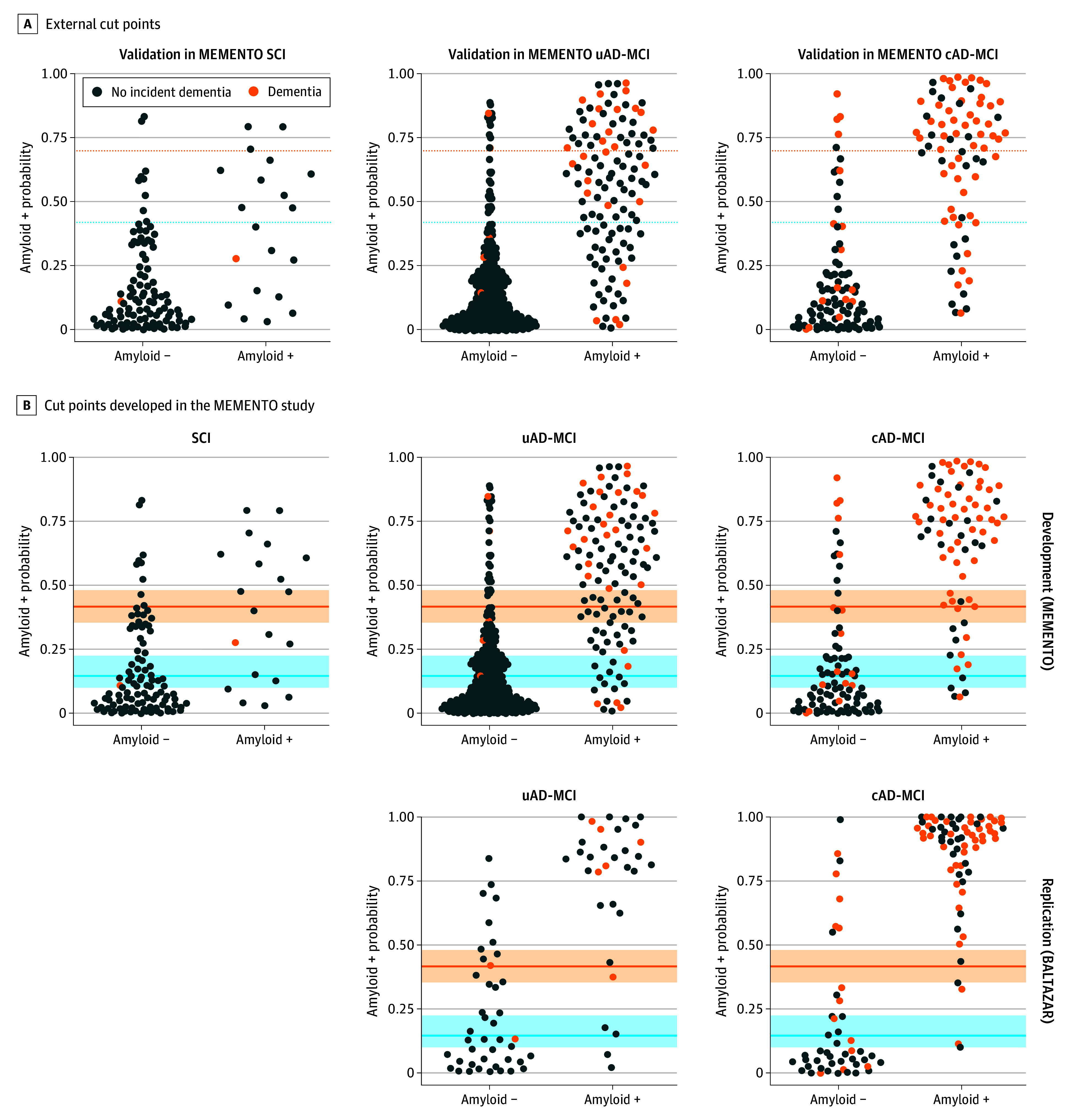
Prediction of Amyloid Positivity (A+) in Different Cognitive Phenotypes Using Blood Phosphorylated Tau 217 (p-Tau217), the MEMENTO and BALTAZAR Studies A, External cut points (validation in the MEMENTO cohort). Colored horizontal lines (42%, 70%) represent the cut points defined in BioFINDER-1 and BioFINDER-2 studies^4^ for a sensitivity and a specificity at 90%. The MEMENTO cohort was used as a validation sample. B, Cut points developed in the MEMENTO study (replication in the BALTAZAR cohort). Colored horizontal lines (15%, 42%) represent the cut points developed in the MEMENTO cohort for a sensitivity and a specificity at 90%. Light orange and light blue bands represent the 95% CI of the cutoff lines. These cut points were then applied on the BALTAZAR replication sample. Each plot represents the probability of being A+ for an individual. Probabilities were derived from a logistic model with age, sex, *APOE* genotype and log(p-tau217) as predictors, fitted independently of the cognitive phenotype and separately for the MEMENTO and BALTAZAR cohorts. Orange dots represent the individuals who developed dementia within the follow-up period. Individuals above the upper cut point line (in orange) were considered A+, and those below the lower cut point line (in blue) were considered amyloid negative (A−). Individuals with a probability between the 2 cut point lines had intermediate probability and would require additional examination (lumbar puncture or amyloid positron emission tomography). AD, Alzheimer disease; BALTAZAR, Biomarker of Amyloid Peptide and Alzheimer’s Disease Risk study; cAD-MCI, MCI with common AD phenotype; MCI, mild cognitive impairment; MEMENTO, The Cohort of Outpatients From French Research Memory Centers in Order to Improve Knowledge on Alzheimer’s Disease and Related Disorders study; SCI, subjective cognitive impairment; uAD-MCI, MCI with an uncommon AD/other phenotype.

Figure 1A shows the estimated probabilities derived from blood p-tau217 models in the MEMENTO cohort with the published cut points to achieve 90% sensitivity and specificity. PPVs varied across cognitive phenotype (60.0%, 90.0%, and 74.5% for SCI, cAD-MCI, and uAD-MCI, respectively), whereas NPVs were high and comparable (ranging from 84.2%-90.2%) (Table 2).

**Table 2.** Determination of Amyloid Status Using Blood Phosphorylated Tau 217 (p-Tau217) in Different Cognitive Phenotypes^a^

Status	Cognitive phenotype, No. (%) [95% CI]			Phenotypes comparisons, *P* value	
	SCI	MCI		Overall	MCI only
		With common AD	With uncommon AD/other		
**MEMENTO: External cut points validation** ^b^					
True positive (PPV)	3 (60.0) [23.1-88.2]	45 (90.0) [78.6-95.7]	41 (74.5) [61.7-84.2]	.06	.04
True negative (NPV)	92 (90.2) [82.9-94.6]	80 (84.2) [75.6-90.2]	335 (89.8) [86.3-92.5]	.27	.12
Undetermined	14 (11.6) [7.0-18.5]	25 (14.7) [10.2-20.8]	57 (11.8) [9.2-14.9]	.31	.87
**MEMENTO: cut points development** ^c^					
True positive (PPV)	10 (52.6) [31.7-72.7]	63 (84.0) [74.1-90.6]	81 (72.3) [63.4-79.8]	.01	.06
True negative (NPV)	66 (93.0) [84.6-97.0]	55 (91.7) [81.9-96.4]	228 (94.6) [91.0-96.8]	.66	.39
Undetermined	31 (25.6) [18.7-34.1]	35 (20.6) [15.2-27.3]	132 (27.2) [23.4-31.3]	.37	.21
**BALTAZAR: cut points replication** ^c^					
True positive (PPV)	NA	68 (89.5) [80.6-94.6]	27 (73.0) [57.0-84.6]	NA	.02
True negative (NPV)	NA	31 (93.9) [80.4-98.3]	23 (92.0) [75.0-97.8]	NA	.77
Undetermined	NA	10 (8.4) [4.6-14.8]	12 (16.2) [9.5-26.2]	NA	.78

Abbreviations: AD, Alzheimer disease; BALTAZAR, Biomarker of Amyloid Peptide and Alzheimer’s Disease Risk study; MCI, mild cognitive impairment; MEMENTO, The Cohort of Outpatients From French Research Memory Centers in Order to Improve Knowledge on Alzheimer’s Disease and Related Disorders study; NA, not applicable; NPV, negative predictive value; PPV, positive predictive value; SCI, subjective cognitive impairment.

^a^ PPV is computed as the proportion of amyloid positivity in the high probability category, and NPV is computed as the proportion of amyloid negativity in the low probability category and undetermined the proportion of individuals between the 2 cut points. Numbers are for a Se = Sp = 90% dual cut point strategy. Details for Se = Sp = 95% strategy are presented in eTable 2 in Supplement 1.

^b^ Cut points: 42%, 70%.

^c^ Cut points: 15%, 42%.

### Cut Points Development in the MEMENTO Cohort and Replication in the BALTAZAR Cohort

Figure 1B shows the estimated probabilities derived from blood p-tau217 models and the cut points to define low, intermediate, and high A+ probabilities in the MEMENTO cohort, with 90% sensitivity and specificity. Individuals with a probability below 15% (95% CI, 10%-23%) were considered at low risk, and those with a probability of at least 42% (95% CI, 36%-48%) were considered at high risk of being A+. PPVs in the MEMENTO cohort were 52.6%, 84.0% and 72.3% for the SCI, cAD-MCI, and uAD-MCI subgroups, respectively, and NPV were 93.0%, 91.7%, and 94.6%, respectively (Table 2). Once these cut points were applied in BALTAZAR, PPVs were 89.5% and 73.0%, and NPVs were 93.9% and 92.0%, in the cAD-MCI and uAD-MCI subgroups, respectively.

With a sensitivity = specificity = 95% cut point strategy, the number of individuals classified as A+ or A− decreases, limiting the statistical power of the analysis with increased CIs length. However, we still observed the pattern of a higher PPV for cAD-MCI than for SCI or uAD-MCI and comparable NPV across phenotypes (eTable 2 in Supplement 1).

### Clinical Prognosis Based on Brain Amyloidosis Probabilities

**Figure 2.**
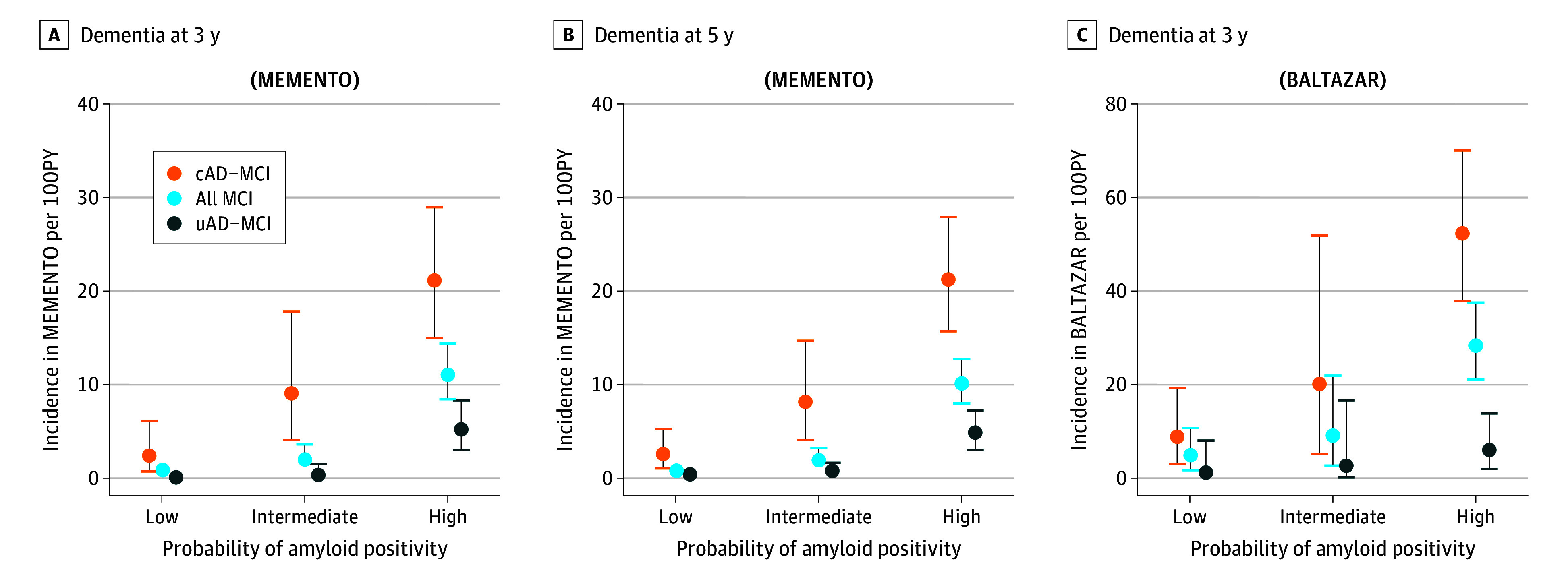
Incidence of Dementia at 3 and 5 Years for Individuals With Mild Cognitive Impairment (MCI) According to Amyloid Positivity Probability The probability of amyloid positivity is provided for cut points developed in The Cohort of Outpatients From French Research Memory Centers in Order to Improve Knowledge on Alzheimer’s Disease and Related Disorders (MEMENTO) study. AD indicates Alzheimer disease; BALTAZAR, Biomarker of Amyloid Peptide and Alzheimer’s Disease Risk study; cAD, common AD phenotype; uAD, uncommon AD/other phenotypes; PY, person-year.

Individuals with cAD-MCI presented a higher rate of incidence dementia compared with those with uAD-MCI. Rates strongly increased with the probability of being A+ (Figure 2). By construction, rates for MCI in general were between cAD and uAD-MCI groups, illustrating the importance of cognitive phenotyping in addition to the determination of amyloid status to estimate clinical progression. This analysis was not performed for individuals with SCI because they presented too few events to provide robust estimations (eFigure in Supplement 1).

### Sensitivity Analysis

Removing *APOE* genotype from the models had a minor impact on discriminative performances and predictive values (eTables 1 and 3 in Supplement 1); eTable 4 in Supplement 1 shows the predictive values based on the criterion standard used to determine amyloid status.

## Discussion

**Figure 3.**
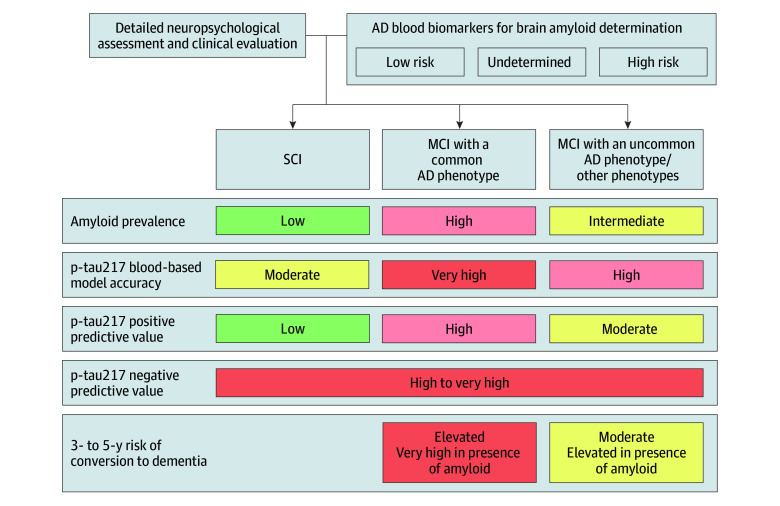
Brain Amyloid Determination Based on Blood Phosphorylated Tau 217 (p-Tau217) Regarding the Cognitive Phenotype of Individuals Attending a Memory Clinic AD indicates Alzheimer disease; MCI, mild cognitive impairment; SCI, subjective cognitive impairment.

In a sample of 776 patients without dementia attending their initial consultation in a memory clinic, we investigated whether detailed cognitive phenotyping was associated with blood p-tau217 interpretation from a diagnostic and prognostic perspective. Aligned with the IWG classification, we found that the proportion of A+ patients with MCI (using criterion-standards amyloid-PET or CSF Aβ42/40) differed according to detailed cognitive phenotyping. We showed the association of differentiating between cAD-MCI vs uAD-MCI with p-tau217 blood-based models AUC. After setting sensitivity and specificity at 90%, we demonstrated that this was markedly associated with the predictive values: although NPVs were comparable across phenotypes, PPVs were higher in patients with cAD-MCI compared with those with uAD-MCI (Figure 3). Moreover, we found that individuals with cAD-MCI had the highest risk of incident dementia, with a 5-fold increase if the probability of being A+ was high according to the p-tau217 measurement. Conversely, individuals with MCI and uAD-MCI experienced a lower risk of incident dementia, even in presence of concurrent brain amyloidosis (Figure 2).

Consistent with the IWG^10^ and European recommendations,^1^ results of this study support the diagnostic approach whereby a detailed cognitive phenotyping should guide the interpretation of biological results. The impact of a rapid clinical evaluation distinguishing MCI from dementia was recently demonstrated on the interpretation of blood biomarkers at the individual level.^31^ However, our results show that this was not optimal in individuals without dementia, considering both diagnostic and prognostic perspectives. Indeed, MCI is underlaid by heterogeneous conditions that blur the interpretation and accuracy of models relying on AD blood biomarkers concentrations. A neuropsychological assessment that clarifies the semiological features of MCI helps to refine the pretest probability of being A+ and thus impacts blood biomarkers predictive values. For common AD phenotypes, ADNC was reported to be 95% in posterior cortical atrophy,^12^ 86% in logopenic primary progressive aphasia,^13^ 50% to 75% in hippocampal amnestic syndrome,^9,14^ and below 30% of cases in uncommon AD phenotype such as corticobasal syndrome or dysexecutive/behavioral variants.^9,15^ Aligned with long-standing MCI subtyping literature,^32^ this study demonstrates the practical clinical impact of differentiating common vs uncommon AD phenotypes.

Individuals with cAD-MCI are often seen in memory clinics^33^ and deserve specific attention given their high risk of cognitive decline and their potential eligibility for antiamyloid immunotherapies.^34,35^ Consequently, a blood biomarker could be considered a valuable alternative to CSF or PET markers because blood biomarkers present good diagnostic performance to rule in and rule out A+ and require a subsequent examination in a small proportion of cases (8%-21% for a sensitivity = specificity = 90% strategy; 22%-37% for a sensitivity = specificity = 95%) (Table 2 and eTable 2 in Supplement 1). However, the added value of complementary examination in individuals with inconclusive AD blood biomarkers needs to be formally explore in future studies, one can hypothesize that CSF or PET evaluation might also lead to intermediate results.

Regarding individuals with uAD-MCI, the use of AD blood biomarkers in clinical practice raises questions. A+ individuals faced an increased risk of incident dementia, whereas A− patients had a risk similar to individuals with SCI. Lower prevalence of brain amyloidosis in this group might alert to the risk of AD overdiagnosis due to false-positive results (low PPV). Furthermore, A+ in patients with uAD phenotype may also be due to copathology, and patients’ symptoms could be primarily driven by other proteinopathies, vascular pathology, or primary psychiatric disorder, for example.^9,10^ Interpreting biomarkers in these patients is particularly challenging, and the initial diagnosis should always be reconsidered based on the patient’s subsequent clinical evolution.

Blood p-tau217 models had moderate performances in predicting brain amyloidosis in individuals with SCI (AUC = 0.78). They present very low dementia conversion rates during the 5-year follow-up in the MEMENTO cohort, whatever their amyloid status, aligning with a recent study that demonstrated that amyloid positivity in itself is not sufficient to identify individuals without cognitive impairment who will develop MCI.^36^ We showed that the risk of false positive is high in individuals with SCI (low PPV); this, therefore, supports the current recommendations of the IWG and the Alzheimer Association not to offer blood biomarker testing in these individuals.^8,10^

### Strengths and Limitations

We acknowledge both strengths and limitations of this work. We used both externally published cut points and developed internal cut points in the MEMENTO cohort to apply them in the BALTAZAR cohort. Although the optimal cut points varied, the conclusion regarding the significant impact of the clinical phenotype on accuracy and predictive values remains the same. The MEMENTO sample comprises nearly 800 well-phenotyped individuals, with a large proportion exhibiting nonmemory cognitive deficits or SCI, addressing the diversity of cases seen in memory clinics. Patients were followed up for 5 years, enabling the study of the risk of dementia in individuals with MCI, a meaningful outcome for patients, families, and caregivers, although too short to study SCI evolution. The baseline neuropsychological data in the MEMENTO and BALTAZAR cohorts allowed us to precisely identify common AD phenotypes (amnestic variant of hippocampal type, posterior cortical atrophy, or logopenic variant primary progressive aphasia) but not to distinguish between uncommon AD phenotypes (eg, corticobasal syndrome, dysexecutive/behavioral variants, nonlogopenic primary progressive aphasias) and other phenotypes (eg, Richardson syndrome, prodromal dementia with Lewy bodies) according to the IWG definition. This limitation required us to gather a heterogeneous group of uncommon/other MCI phenotypes. The method used to determine the amyloid status could be based on CSF or PET scan, and the assays used to determine p-tau concentrations differed between the 2 studies. This heterogeneity reflects the real-world practices, and results remained consistent in the 2 samples. Finally, we include *APOE* genotype in the main analysis to the sake of reproducibility. However, because systematic *APOE* genotyping raises practical and ethical questions,^37^ we hypothesize that AD blood biomarkers concentrations will essentially be interpreted blinded to *APOE* status (eTable 2 in Supplement 1).

## Conclusions

In conclusion, in this cohort study, differentiating between MCI subtypes in individuals attending memory clinics was significantly associated with the diagnostic performance of blood p-tau217 models to determine brain amyloidosis in patients’ clinical management. Our analyses provide experimental foundations for the recent recommendations of the Global CEO Initiative on AD, emphasizing that all biomarkers tests’ predictive values vary according to the pretest probability of amyloid pathology, and stress the need to incorporate detailed cognitive phenotyping to refine this pretest probability.^16,38^ Individuals with MCI presenting a common AD phenotype might benefit the most from these blood biomarkers to avoid invasive or expensive examination and provide relevant insights into their cognitive prognosis.

## Notes

Supplement 1. **eFigure.** Kaplan-Meier Curves for Incident Dementia According to Cognitive Phenotype and Amyloid Status **eTable 1.** Discriminative Performances to Predict the Amyloid Positivity Using p-Tau217 Blood-Based Models **eTable 2.** Determination of Amyloid Status Using Blood p-Tau217 in Different Cognitive Phenotypes Using a Se=Sp=95% Cut Points Definition **eTable 3.** Determination of Amyloid Status Using Blood p-Tau217 Without *APOE* in Different Cognitive Phenotypes **eTable 4.** Predictive Values for Determination of CSF-Based and PET-Based Amyloid Status Using p-Tau217 Blood-Based Models (Se=Sp=90%)

Supplement 2. **Nonauthor Collaborators.** The MEMENTO and BALTAZAR Study Groups

Supplement 3.
Data Sharing Statement.
