# Supplementary material for: Cognitive Phenotyping and Interpretation of Alzheimer Blood Biomarkers
Source: JAMA Neurol. 2025 Apr 4;82(5):506–15. doi: 10.1001/jamaneurol.2025.0142 (PMC11971688; doi:10.1001/jamaneurol.2025.0142)
Supplement: Supplement 1. — eFigure. Kaplan-Meier Curves for Incident Dementia According to Cognitive Phenotype and Amyloid Status eTable 1. Discriminative Performances to Predict the Amyloid Positivity Using p-Tau217 Blood-Based Models eTable 2. Determination of Amyloid Status Using Blood p-Tau217 in Different Cognitive Phenotypes Using a Se=Sp=95% Cut Points Definition eTable 3. Determination of Amyloid Status Using Blood p-Tau217 Without APOE in Different Cognitive Phenotypes eTable 4. Predictive Values for Determination of CSF-Based and PET-Based Amyloid Status Using p-Tau217 Blood-Based Models (Se=Sp=90%) [file jamaneurol-e250142-s001.pdf]

Supplementary Online Content

Bouteloup V, Villain N, Vidal JS, et al; MEMENTO and BALTAZAR Study Groups. Cognitive phenotyping and interpretation of Alzheimer blood biomarkers. *JAMA Neurol*. Published online April 4, 2025. doi:10.1001/jamaneurol.2025.0142

eFigure. Kaplan-Meier Curves for Incident Dementia According to Cognitive Phenotype and Amyloid Status

eTable 1. Discriminative Performances to Predict the Amyloid Positivity Using p-Tau217 Blood-Based Models

eTable 2. Determination of Amyloid Status Using Blood p-Tau217 in Different Cognitive Phenotypes Using a Se=Sp=95% Cut Points Definition

eTable 3. Determination of Amyloid Status Using Blood p-Tau217 Without *APOE* in Different Cognitive Phenotypes

eTable 4. Predictive Values for Determination of CSF-Based and PET-Based Amyloid Status Using p-Tau217 Blood-Based Models (Se=Sp=90%)

This supplementary material has been provided by the authors to give readers additional information about their work.

© 2025 Bouteloup V et al. *JAMA Neurology*.

A. MEMENTO

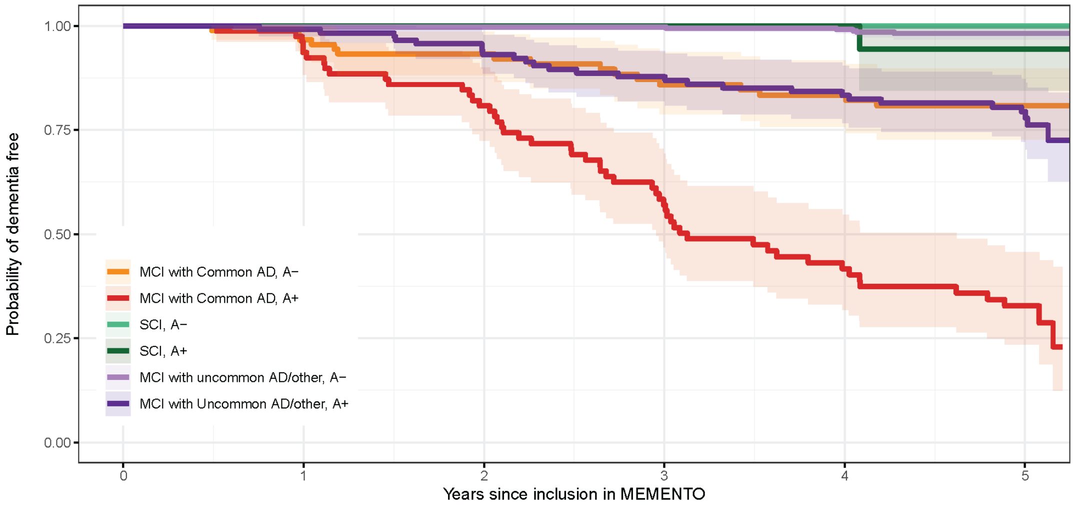

Number at risk (number of events)

0	1	2	3	4	5
92 (0)	86 (3)	80 (6)	69 (12)	66 (14)	44 (16)
78 (0)	73 (5)	63 (15)	42 (33)	29 (44)	14 (50)
101 (0)	96 (0)	96 (0)	93 (0)	92 (0)	66 (0)
20 (0)	20 (0)	20 (0)	19 (0)	19 (0)	13 (1)
366 (0)	352 (1)	346 (1)	336 (1)	320 (3)	235 (6)
119 (0)	115 (1)	107 (8)	99 (14)	91 (19)	52 (23)

Years since inclusion in MEMENTO

B. BALTAZAR

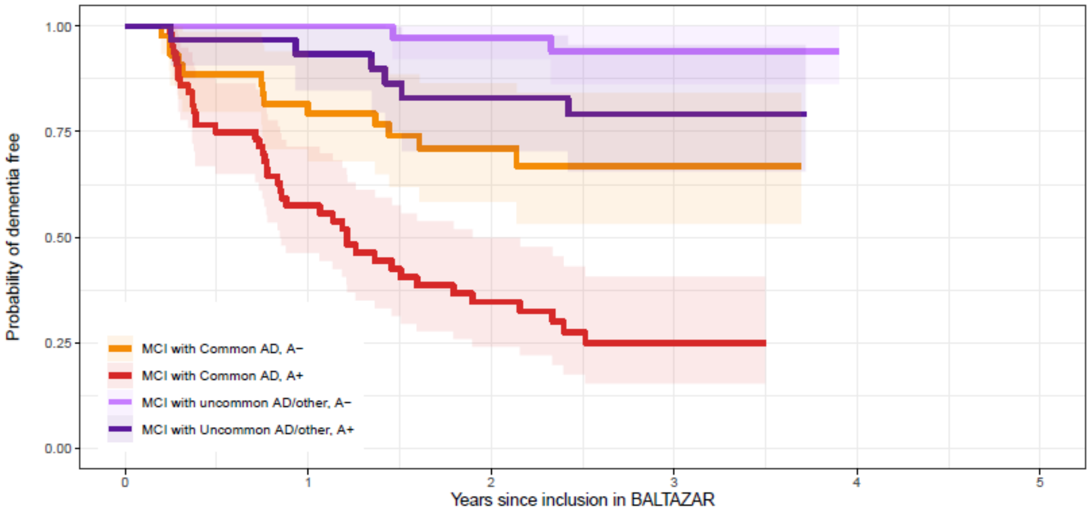

Number at risk (number of events)

0	1	2	3	4	5
47 (0)	33 (9)	23 (12)	11 (13)	0 (13)	0 (13)
72 (0)	33 (26)	16 (38)	9 (42)	0 (42)	0 (42)
42 (0)	37 (0)	35 (1)	27 (2)	0 (2)	0 (2)
32 (0)	27 (2)	24 (5)	17 (6)	0 (6)	0 (6)

Years since inclusion in BALTAZAR

eFigure. Kaplan-Meier curves for incident dementia according to cognitive phenotype and amyloid status. The MEMENTO and BALTAZAR studies

AD: Alzheimer's disease; MCI: Mild cognitive impairment, SCI: Subjective Cognitive Impairment

© 2025 Bouteloup V et al. *JAMA Neurology*.

eTable 1. Discriminative performances to predict the amyloid positivity using p-tau217 blood-based models. The MEMENTO and BALTAZAR studies

	Prediction based on p-tau217 and *APOE*		Prediction based on p-tau217 without *APOE*	
AUC [95%CI]	MEMENTO	BALTAZAR	MEMENTO	BALTAZAR
Overall	0.88 [0.85;0.91]	0.94 [0.90;0.97]	0.86 [0.84;0.89]	0.92 [0.88;0.96]
By clinical phenotype[a]				
SCI	0.78 [0.66;0.89]	-	0.76 [0.64;0.88]	-
MCI with Common AD	0.91 [0.86;0.95]	0.95 [0.91;0.99]	0.88 [0.83;0.93]	0.93 [0.88;0.98]
MCI with Uncommon AD/other	0.87 [0.84;0.91]	0.90 [0.82;0.97]	0.86 [0.82;0.9]	0.87 [0.79;0.95]

Amyloid positivity predictions were computed with logistic regression models including age, sex, and log(p-tau217) and +/- *APOE* genotype, independently of the cognitive phenotype, and separately for the MEMENTO and BALTAZAR samples.

a) AUC by phenotype are derived from the unique overall model and are presented for illustrative purpose

AD: Alzheimer's disease, *APOE*: Apolipoprotein E, AUC: Area under the curve, CI: Confidence Interval, MCI: Mild cognitive impairment, SCI: Subjective Cognitive Impairment

© 2025 Bouteloup V et al. *JAMA Neurology*.

eTable 2. Determination of amyloid status using blood p-tau217 in different cognitive phenotypes using a Se=Sp=95% cut points definition. The MEMENTO and BALTAZAR studies

	Cognitive phenotype						Phenotypes comparisons (p-values)	
	SCI		MCI with Common AD		MCI with Uncommon AD/other			
	n (%)	[95%CI]	n (%)	[95%CI]	n (%)	[95%CI]	Overall	MCI only
**MEMENTO: external cutpoints validation[a]**								
True positive (PPV)	0 (0)	-	29 (90.6)	[75.8;96.8]	23 (69.7)	[52.7;82.6]	0.004	0.035
True negative (NPV)	78 (89.7)	[81.5;94.5]	74 (87.1)	[78.3;92.6]	314 (92.6)	[89.3;95.0]	0.23	0.10
Undetermined	32 (26.4)	[19.4;34.9]	53 (31.2)	[24.7;38.5]	113 (23.3)	[19.8;27.3]	0.002	0.26
**MEMENTO: Cutpoints development[b]**								
True positive (PPV)	6 (66.7)	[35.4;87.9]	55 (85.9)	[75.4;92.4]	57 (76)	[65.2;84.2]	0.21	0.14
True negative (NPV)	45 (93.8)	[83.2;97.9]	41 (95.3)	[84.5;98.7]	168 (96)	[92.0;98.0]	0.80	0.85
Undetermined	64 (52.9)	[44.0;61.6]	63 (37.1)	[30.2;44.5]	235 (48.5)	[44;52.9]	0.096	0.11
**BALTAZAR: Cutpoints replication[b]**								
True positive (PPV)			64 (92.8)	[84.1;96.9]	26 (86.7)	[70.3;94.7]		0.33
True negative (NPV)			24 (100)	[86.2;100]	16 (88.9)	[67.2;96.9]		0.094
Undetermined			26 (21.8)	[15.4;30.1]	26 (35.1)	[25.2;46.5]		0.19

AD: Alzheimer's disease, CI: Confidence Interval, MCI: Mild cognitive impairment, PPV: Positive predictive value, NPV: negative predictive value, SCI: Subjective Cognitive Impairment, Se: Sensitivity, Sp: Specificity

Positive predictive value (PPV) are computed as the proportion of amyloid+ in the high probability category, negative predictive value (NPV) as the proportion of amyloid- in the low probability category and undetermined the proportion of individuals between the two cutpoints.

a) cutpoints: 31%, 80% b) cutpoints: 8%, 61%

© 2025 Bouteloup V et al. *JAMA Neurology*.

eTable 3. Determination of amyloid status using blood p-tau217 without *APOE* in different cognitive phenotypes (Se=Sp=90%). The MEMENTO and BALTAZAR studies

	Cognitive phenotype						Phenotypes comparisons (p-values)	
	SCI		MCI with Common AD		MCI with Uncommon AD/other			
	n (%)	[95%CI]	n (%)	[95%CI]	n (%)	[95%CI]	Overall	MCI only
**MEMENTO: Cutpoints development[a]**								
True positive (PPV)	9 (45)	[25.8;65.8]	58 (81.7)	[71.2;89.0]	78 (69.6)	[60.6;77.4]	0.005	0.069
True negative (NPV)	54 (91.5)	[81.6;96.3]	56 (91.8)	[82.2;96.4]	221 (94.8)	[91.2;97.0]	0.50	0.36
Undetermined	42 (34.7)	[26.8;43.5]	38 (22.4)	[16.7;29.2]	140 (28.9)	[25.0;33.1]	0.017	0.017
**BALTAZAR: Cutpoints replication[a]**								
True positive (PPV)			68 (89.5)	[80.6;94.6]	25 (71.4)	[54.9;83.7]		0.017
True negative (NPV)			29 (93.5)	[79.3;98.2]	20 (90.9)	[72.2;97.5]		0.72
Undetermined			12 (10.1)	[5.9;16.8]	17 (23)	[14.9;33.7]		0.43

AD: Alzheimer's disease, CI: Confidence Interval, MCI: Mild cognitive impairment, PPV: Positive predictive value; NPV: negative predictive value, SCI: Subjective Cognitive Impairment, Se: Sensitivity, Sp: Specificity

Positive predictive value (PPV) are computed as the proportion of amyloid+ in the high probability category, negative predictive value (NPV) as the proportion of amyloid- in the low probability category and undetermined the proportion of individuals between the two cutpoints.

a) cutpoints: 15%, 45%

© 2025 Bouteloup V et al. *JAMA Neurology*.

eTable 4. Predictive values for determination of CSF-based and PET-based amyloid status using p-tau217 blood-based models (Se=Sp=90%). The MEMENTO and BALTAZAR studies

	Positive predictive value [95%CI]		negative predictive value [95%CI]	
Amyloid status definition	CSF-based	PET-based	CSF-based	PET-based
SCI	85.7 [48.7 ;99.3]	31.2 [14.2 ;55.6]	87.5 [69.0 ;95.7]	95.9 [86.3 ;98.9]
MCI with Common AD	86.4 [73.3 ;93.6]	87.0 [67.9 ;95.5]	90.9 [76.4 ;96.9]	94.6 [82.3 ;98.5]
MCI with Uncommon AD/other	66.7 [51.6 ;79.0]	74.0 [63.3 ;82.5]	92.6 [84.8 ;96.6]	96.5 [93.0 ;98.3]

CI: Confidence Interval, CSF: Cerebrospinal Fluid, PET: positron emission tomography, AD: Alzheimer’s disease, MCI: Mild cognitive impairment, SCI: Subjective Cognitive Impairment, Se: Sensitivity, Sp: Specificity

Cutpoints were defined independently for individuals with CSF or PET evaluation regardless of the cognitive phenotype.

© 2025 Bouteloup V et al. *JAMA Neurology*.
