# Supplementary material for: Cognitive Phenotyping and Interpretation of Alzheimer Blood Biomarkers
Source: JAMA Neurol. 2025 Apr 4;82(5):506–15. doi: 10.1001/jamaneurol.2025.0142 (PMC11971688; doi:10.1001/jamaneurol.2025.0142)
Supplement: Supplement 2. — Nonauthor Collaborators. The MEMENTO and BALTAZAR Study Groups [file jamaneurol-e250142-s002.pdf]

Supplemental Online Content: Nonauthor Collaborators
*First name, last name, and suffix (if applicable) are required and will appear in PubMed.

*Group Name(s): The MEMENTO and the BALTAZAR Study groups

*First Name and Middle In	*Last Name	*Suffix (eg, Jr, III)	Academic D	Institution	Location (city, stat	Role or Contribution,	Group (if more than 1 Group listed in the byline) and/or Subgroup (eg, Steering Committee)
Isabelle	Addra			Coordinating Centre, Inserm CIC-1401 Clinical Epidemiology, CHU de Bordeaux, F-33000, Bordeaux, France			The MEMENTO Study Group
Michèle	Allard			Memory Resource and Research Centre of Bordeaux, CHU de Bordeaux, Hôpital Xavier Arnozan, F-33000, Bordeaux, France			The MEMENTO Study Group
Sandrine	Andrieu			Memory Resource and Research Centre of Toulouse, CHU de Toulouse, Hôpital La Grave-Casselardit, F-31000, Toulouse, France			The MEMENTO Study Group
Pierre	Anthony			Memory Resource and Research Centre of Colmar, Hôpitaux Civils de Colmar, F-68000, Colmar, France			The MEMENTO Study Group
Christine	Astier			Memory Resource and Research Centre of Strasbourg, Hôpitaux Universitaires de Strasbourg, F-67000, Strasbourg, France			The MEMENTO Study Group
Alexandre	Augier			Memory Clinic, Hôpital Avicenne, AP-HP, Hôpitaux Universitaires Paris-Seine-Saint-Denis, F-93009, Bobigny, France			The MEMENTO Study Group
Nicolas	Auguste			Memory Resource and Research Centre of Saint-Etienne, CHU de Saint-Etienne, Hôpital de la Charité, F-42000, Saint-Etienne, France			The MEMENTO Study Group
Sophie	Auriacombe			Memory Resource and Research Centre of Bordeaux, CHU de Bordeaux, Hôpital Pellegrin, F-33000, Bordeaux, France			The MEMENTO Study Group
John	Avet			Memory Resource and Research Centre of Saint-Etienne, CHU de Saint-Etienne, Hôpital Nord, F-42000, Saint-Etienne, France			The MEMENTO Study Group
Chabha	Azouani			Institute of Memory and Alzheimer's Disease (IM2A), Centre for NeuroImaging Research (CENIR), Brain and Spine Institute (ICM), UMR S 1127, Departm			The MEMENTO Study Group
Olivier	Bailon			Memory Clinic, Hôpital Avicenne, AP-HP, Hôpitaux Universitaires Paris-Seine-Saint-Denis, F-93009, Bobigny, France			The MEMENTO Study Group
Anna-Chloé	Balageas			Memory Resource and Research Centre of Center Region, CHRU de Tours, Hôpital Bretonneau, F-37000, Tours, France			The MEMENTO Study Group
Fabrice-Guy	Barral			Memory Resource and Research Centre of Saint-Etienne, CHU de Saint-Etienne, Hôpital Nord, F-42000, Saint-Etienne, France			The MEMENTO Study Group
Jean	Barré			Memory Resource and Research Centre of Angers, CHU d'Angers, F-49000, Angers			The MEMENTO Study Group
Annick	Barthelaix			Memory Resource and Research Centre of Angers, CHU d'Angers, F-49000, Angers			The MEMENTO Study Group
Catherine	Bayle			Memory Resource and Research Centre of Paris Broca, AP-HP, Paris, France			The MEMENTO Study Group
Olivier	Beauchet			Memory Resource and Research Centre of Angers, CHU d'Angers, F-49000, Angers			The MEMENTO Study Group
Serge	Belliard			Memeory Ressource and Research Centre of Rennes, CHU Rennes, Hôpital Pontchaillou, Rennes F-35000, Rennes			The MEMENTO Study Group
Catherine	Belin			Memory Clinic, Hôpital Avicenne, AP-HP, Hôpitaux Universitaires Paris-Seine-Saint-Denis, F-93009, Bobigny, France			The MEMENTO Study Group
Samia	Belkacem			Institute of Memory and Alzheimer's Disease (IM2A), Centre for NeuroImaging Research (CENIR), Brain and Spine Institute (ICM), UMR S 1127, Departm			The MEMENTO Study Group
Hugo	Bertin			Automated Image Acquisition Processing Center – CATI, Functional Imaginf Laboratory – UMR 678 / Pierre et Marie Curie University – 75634 Paris Cede			The MEMENTO Study Group
Douraied	Ben Salem			Memory Resource and Research Centre of Brest, CHRU de Brest, F-29000, Brest, France			The MEMENTO Study Group
Karim	Bennys			Memory Resource and Research Centre of Montpellier, CHU de Montpellier, Hôpital Gui de Chauliac, F-34000, Montpellier, France			The MEMENTO Study Group
Géraldine	Bera			Laboratoire d'Imagerie Biomédicale, Sorbonne Universités, UPMC Univ Paris 06, Inserm U1146, CNRS UMR 7371, France NeuroSpin, I2BM, Commissar			The MEMENTO Study Group
Eric	Berger			Memory Resource and Research Centre of Besançon, CHU de Besançon, Hôpital Jean Minjoz, Hôpital Saint-Jacques, F-25000, Besançon, France			The MEMENTO Study Group
Marc G	Berger			Memory Resource and Research Centre of Clermont-Ferrand, CHU de Clermont-Ferrand, F-63000, Clermont-Ferrand, France			The MEMENTO Study Group
Emilie	Bergouin			Memory Resource and Research Centre of Dijon, CHU Dijon Bourgogne, Hôpital du Bocage, Hôpital de Champmaillot, F-21000, Dijon, France			The MEMENTO Study Group
François	Bertin-Hugault			Memory Resource and Research Centre of Lyon, Hospices Civils de Lyon, Hôpital des Charpennes, F-69000, Lyon, France			The MEMENTO Study Group
Guillaume	Bertrand			Memory Clinic, Hôpital Avicenne, AP-HP, Hôpitaux Universitaires Paris-Seine-Saint-Denis, F-93009, Bobigny, France			The MEMENTO Study Group
François-Xavier	Bertrand			Memory Resource and Research Centre of Nantes, CHU de Nantes, F-44000, Nantes, France			The MEMENTO Study Group
Catherine	Beze			Memory Resource and Research Centre of Center Region, CHRU de Tours, Hôpital Bretonneau, F-37000, Tours, France			The MEMENTO Study Group
Valérie	Boilet			Coordinating Centre, Inserm CIC-1401 Clinical Epidemiology, CHU de Bordeaux, F-33000, Bordeaux, France			The MEMENTO Study Group
Stéphanie	Bombois			Institute of Memory and Alzheimer's Disease (IM2A), Brain and Spine Institute (ICM), UMR S 1127, Department of Neurology, AP-HP, Pitié-Salpêtrière			The MEMENTO Study Group
Alain	Bonafé			Memory Resource and Research Centre of Montpellier, CHU de Montpellier, Montpellier, France			The MEMENTO Study Group
Yasmina	Boudali			Memory Resource and Research Centre of Paris Broca, AP-HP, Paris, France			The MEMENTO Study Group
Hatem	Bouhladour			Memory Resource and Research Centre of Besançon, CHU de Besançon, Hôpital Jean Minjoz, Hôpital Saint-Jacques, F-25000, Besançon, France			The MEMENTO Study Group
Clémence	Boully			Memory Resource and Research Centre of Paris Broca, AP-HP, Paris, France			The MEMENTO Study Group
Isabelle	Bourdel-Marchasson			Memory Resource and Research Centre of Bordeaux, CHU de Bordeaux, Hôpital Xavier Arnozan, F-33000, Bordeaux, France			The MEMENTO Study Group
Vincent	Bouteloup			Coordinating Centre, Inserm CIC-1401 Clinical Epidemiology, CHU de Bordeaux, F-33000, Bordeaux, France			The MEMENTO Study Group
Claire	Boutet			Institute of Memory and Alzheimer's Disease (IM2A), Centre for NeuroImaging Research (CENIR), Brain and Spine Institute (ICM), UMR S 1127, Departm			The MEMENTO Study Group
Christophe	Bouvier			Coordinating Centre, Inserm CIC-1401 Clinical Epidemiology, CHU de Bordeaux, F-33000, Bordeaux, France			The MEMENTO Study Group
Laïla	Bouzid			Coordinating Centre, Inserm CIC-1401 Clinical Epidemiology, CHU de Bordeaux, F-33000, Bordeaux, France			The MEMENTO Study Group
Serge	Bracard			Memory Resource and Research Centre of Nancy, CHU de Nancy, F-54000, Nancy, France			The MEMENTO Study Group
Antoine	Brangier			Memory Resource and Research Centre of Angers, CHU d'Angers, F-49000, Angers			The MEMENTO Study Group
Pierre-Yves	Brillet			Memory Clinic, Hôpital Avicenne, AP-HP, Hôpitaux Universitaires Paris-Seine-Saint-Denis, F-93009, Bobigny, France			The MEMENTO Study Group
Laure	Caillard			Memory Resource and Research Centre of Paris Broca, AP-HP, Paris, France			The MEMENTO Study Group
Fabienne	Calvas			Memory Resource and Research Centre of Toulouse, CHU de Toulouse, Hôpital Purpan, F-31000, Toulouse, France			The MEMENTO Study Group
Agnès	Camus			Memory Resource and Research Centre of Dijon, CHU Dijon Bourgogne, Hôpital du Bocage, Hôpital de Champmaillot, F-21000, Dijon, France			The MEMENTO Study Group
Vincent	Camus			Memory Resource and Research Centre of Center Region, CHRU de Tours, Hôpital Bretonneau, F-37000, Tours, France			The MEMENTO Study Group
Sandrine	Canaple			Memory Resource and Research of Amiens, CHU Amiens Picardie, F-80000, Amiens, France			The MEMENTO Study Group
Alexandre	Cantan			Coordinating Centre, Inserm CIC-1401 Clinical Epidemiology, CHU de Bordeaux, F-33000, Bordeaux, France			The MEMENTO Study Group
Antoine	Carpentier			Memory Clinic, Hôpital Avicenne, AP-HP, Hôpitaux Universitaires Paris-Seine-Saint-Denis, F-93009, Bobigny, France			The MEMENTO Study Group
Pascaline	Cassagnaud			Memory Resource and Research Centre of Lille, CHRU de Lille, Hôpital Roger Salengro, F-59000, Lille, France			The MEMENTO Study Group
Françoise	Cattin			Memory Resource and Research Centre of Besançon, CHU de Besançon, Hôpital Jean Minjoz, Hôpital Saint-Jacques, F-25000, Besançon, France			The MEMENTO Study Group
Ludivine	Chamard			Memory Resource and Research Centre of Besançon, CHU de Besançon, Hôpital Jean Minjoz, Hôpital Saint-Jacques, F-25000, Besançon, France			The MEMENTO Study Group
Stéphane	Chanalet			Memory Resource and Research Centre of Nice, CHU de Nice, Hôpital Pasteur, F-06100, Nice, France			The MEMENTO Study Group
Mathieu	Chastan			Memory Resource and Research Centre of Rouen, CLCC Henri Becquerel, Rouen, France			The MEMENTO Study Group
Sophie	Chauvelier			Memory Resource and Research Centre of Paris Broca, AP-HP, Paris, France			The MEMENTO Study Group
Valérie	Chauvire			Memory Resource and Research Centre of Angers, CHU d'Angers, F-49000, Angers			The MEMENTO Study Group
Geneviève	Chêne			Coordinating Centre, Inserm CIC-1401 Clinical Epidemiology, CHU de Bordeaux, F-33000, Bordeaux, France			The MEMENTO Study Group
Samia	Cheriet			Memory Resource and Research Centre of Toulouse, CHU de Toulouse, Hôpital Purpan, F-31000, Toulouse, France			The MEMENTO Study Group
Marie	Chupin			Institute of Memory and Alzheimer's Disease (IM2A), Centre for NeuroImaging Research (CENIR), Brain and Spine Institute (ICM), UMR S 1127, Departm			The MEMENTO Study Group
Anthony	Clotagatide			Memory Resource and Research Centre of Saint-Etienne, CHU de Saint-Etienne, Hôpital Nord, F-42000, Saint-Etienne, France			The MEMENTO Study Group
Emmanuel	Cognat			Memory Resource and Research Centre of Paris Nord, AP-HP, Paris, France			The MEMENTO Study Group
Lora	Cohen			Memory Resource and Research Centre of Grenoble, CHU de Grenoble Alpes, Grenoble, France			The MEMENTO Study Group
Jean-Marc	Constans			Memory Resource and Research of Amiens, CHU Amiens Picardie, F-80000, Amiens, France			The MEMENTO Study Group

*First name, last name, and suffix (if applicable) are required and will appear in PubMed.

*First Name and Middle In	*Last Name	*Suffix (eg, Jr, III)	Academic D	Institution	Location (city, stat	Role or Contribution,	Group (if more than 1 Group listed in the byline) and/or Subgroup (eg, Steering Committee)
Marie-Hélène	Coste			Memory Resource and Research Centre of Lyon, Hospices Civils de Lyon, Hôpital des Charpennes, F-69000, Lyon, France			The MEMENTO Study Group
Jean-Philippe	Cottier			Memory Resource and Research Centre of Center Region, CHRU de Tours, Hôpital Bretonneau, F-37000, Tours, France			The MEMENTO Study Group
François	Cotton			Memory Resource and Research Centre of Lyon, Hospices Civils de Lyon, Hôpital des Charpennes, F-69000, Lyon, France			The MEMENTO Study Group
Isabelle	Couret			Memory Resource and Research Centre of Montpellier, CHU de Montpellier, Hôpital Gui de Chauliac, F-34000, Montpellier, France			The MEMENTO Study Group
Olivier-François	Couturier			Memory Resource and Research Centre of Angers, CHU d'Angers, F-49000, Angers			The MEMENTO Study Group
Pascale	Cowppli-Bony			Memory Resource and Research Centre of Bordeaux, CHU de Bordeaux, Hôpital Pellegrin, F-33000, Bordeaux, France			The MEMENTO Study Group
Véronique	Cressot			Memory Resource and Research Centre of Bordeaux, CHU de Bordeaux, Hôpital Xavier Arnozan, F-33000, Bordeaux, France			The MEMENTO Study Group
Benjamin	Crétin			Memory Resource and Research Centre of Strasbourg, Hôpitaux Universitaires de Strasbourg, F-67000, Strasbourg, France			The MEMENTO Study Group
Keren	Danaila			Memory Resource and Research Centre of Lyon, Hospices Civils de Lyon, Hôpital des Charpennes, F-69000, Lyon, France			The MEMENTO Study Group
Jacques	Darcourt			Memory Resource and Research Centre of Nice, CLCC Antoine Lacassagne, Nice, France			The MEMENTO Study Group
Jean-François	Dartigues			Memory Resource and Research Centre of Bordeaux, CHU de Bordeaux, Hôpital Pellegrin, F-33000, Bordeaux, France			The MEMENTO Study Group
Ana-Maria	Dascalita			Memory Resource and Research Centre of Saint-Etienne, CHU de Saint-Etienne, Hôpital de la Charité, F-42000, Saint-Etienne, France			The MEMENTO Study Group
Renaud	David			Memory Resource and Research Centre of Nice, CHU de Nice, Institut Claude Pompidou, F-06100, Nice, France			The MEMENTO Study Group
Samuel	David			Automated Image Acquisition Processing Center – CATI, Functional Imaginf Laboratory – UMR 678 / Pierre et Marie Curie University – 75634 Paris Cede			The MEMENTO Study Group
Xavier	De Petigny			Memory Resource and Research Centre of Strasbourg, Hôpitaux Universitaires de Strasbourg, F-67000, Strasbourg, France			The MEMENTO Study Group
Delphine	De Verbizier-Lonjon			Memory Resource and Research Centre of Montpellier, CHU de Montpellier, Hôpital Gui de Chauliac, F-34000, Montpellier, France			The MEMENTO Study Group
Marielle	Decousus			Memory Resource and Research Centre of Saint-Etienne, CHU de Saint-Etienne, Hôpital Nord, F-42000, Saint-Etienne, France			The MEMENTO Study Group
Isabelle	Defouilloy			Memory Resource and Research of Amiens, CHU Amiens Picardie, F-80000, Amiens, France			The MEMENTO Study Group
Christine	Delmaire			Memory Resource and Research Centre of Lille, CHRU de Lille, Hôpital Roger Salengro, F-59000, Lille, France			The MEMENTO Study Group
Julien	Delrieu			Memory Resource and Research Centre of Toulouse, CHU de Toulouse, Hôpital La Grave-Casselardit, F-31000, Toulouse, France			The MEMENTO Study Group
Catherine	Demuyinck			Memory Resource and Research Centre of Strasbourg, Hôpitaux Universitaires de Strasbourg, F-67000, Strasbourg, France			The MEMENTO Study Group
Vincent	Deramecourt			Memory Resource and Research Centre of Lille, CHRU de Lille, Hôpital Roger Salengro, F-59000, Lille, France			The MEMENTO Study Group
Hervé	Deramond			Memory Resource and Research of Amiens, CHU Amiens Picardie, F-80000, Amiens, France			The MEMENTO Study Group
Thomas	Desmidt			Memory Resource and Research Centre of Center Region, CHRU de Tours, Hôpital Bretonneau, F-37000, Tours, France			The MEMENTO Study Group
Marie-Dominique	Desruet			Memory Resource and Research Centre of Grenoble, CHU de Grenoble Alpes, Grenoble, France			The MEMENTO Study Group
Julien	Detour			Memory Resource and Research Centre of Strasbourg, Hôpitaux Universitaires de Strasbourg, F-67000, Strasbourg, France			The MEMENTO Study Group
Agnès	Devendeville			Memory Resource and Research of Amiens, CHU Amiens Picardie, F-80000, Amiens, France			The MEMENTO Study Group
Mira	Didic			Memory Resource and Research Centre of Marseille, CHU de Marseille, Hôpital La Timone, F-13000, Marseille, France			The MEMENTO Study Group
Sonia	Djober			Institute of Memory and Alzheimer's Disease (IM2A), Centre for NeuroImaging Research (CENIR), Brain and Spine Institute (ICM), UMR S 1127, Departn			The MEMENTO Study Group
Maritchu	Doireau			Memory Resource and Research Centre of Bordeaux, CHU de Bordeaux, Hôpital Pellegrin, F-33000, Bordeaux, France			The MEMENTO Study Group
Antonio	Dos Santos			Institute of Memory and Alzheimer's Disease (IM2A), Brain and Spine Institute (ICM), UMR S 1127, Department of Neurology, AP-HP, Pitié-Salpêtrière			The MEMENTO Study Group
Patrice	Douillet			Memory Resource and Research Centre of Montpellier, CHU de Montpellier, Hôpital Gui de Chauliac, F-34000, Montpellier, France			The MEMENTO Study Group
Foucaud	Du Boisgueheneuc			Memory Resource and Research Centre of Poitiers, CHU de Poitiers, Hôpital de La Milétrie, F-86000, Poitiers, France			The MEMENTO Study Group
Delphine	Dubail			Memory Resource and Research Centre of Paris Broca, AP-HP, Paris, France			The MEMENTO Study Group
Laure	Ducroq-Ducastaing			Memory Resource and Research Centre of Brest, CHRU de Brest, F-29000, Brest, France			The MEMENTO Study Group
Carole	Dufouil			Coordinating Centre, Inserm CIC-1401 Clinical Epidemiology, CHU de Bordeaux, F-33000, Bordeaux, France			The MEMENTO Study Group
Cynthia	Dumoulin			Coordinating Centre, Inserm CIC-1401 Clinical Epidemiology, CHU de Bordeaux, F-33000, Bordeaux, France			The MEMENTO Study Group
Julien	Dumurgier			Memory Resource and Research Centre of Paris Nord, AP-HP, Paris, France			The MEMENTO Study Group
Diane	Dupuy			Memory Resource and Research of Amiens, CHU Amiens Picardie, F-80000, Amiens, France			The MEMENTO Study Group
Emmanuelle	Duron			Memory Resource and Research Centre of Paris Broca, AP-HP, Paris, France			The MEMENTO Study Group
Inna	Dygai-Cochet			Memory Resource and Research Centre of Dijon, CLCC Georges François Leclerc, Dijon, France			The MEMENTO Study Group
Véronique	Eder			Memory Clinic, Hôpital Avicenne, AP-HP, Hôpitaux Universitaires Paris-Seine-Saint-Denis, F-93009, Bobigny, France			The MEMENTO Study Group
Fouzia	El Mountassir			Institute of Memory and Alzheimer's Disease (IM2A), Centre for NeuroImaging Research (CENIR), Brain and Spine Institute (ICM), UMR S 1127, Departn			The MEMENTO Study Group
Stéphane	Epelbaum			Institute of Memory and Alzheimer's Disease (IM2A), Brain and Spine Institute (ICM), UMR S 1127, Department of Neurology, AP-HP, Pitié-Salpêtrière			The MEMENTO Study Group
Julie	Erraud			Coordinating Centre, Inserm CIC-1401 Clinical Epidemiology, CHU de Bordeaux, F-33000, Bordeaux, France			The MEMENTO Study Group
Frédérique	Etcharry-Bouyx			Memory Resource and Research Centre of Angers, CHU d'Angers, F-49000, Angers			The MEMENTO Study Group
Daniel	Fagret			Memory Resource and Research Centre of Grenoble, CHU de Grenoble Alpes, Grenoble, France			The MEMENTO Study Group
Catherine	Faisant			Memory Resource and Research Centre of Toulouse, CHU de Toulouse, Hôpital La Grave-Casselardit, F-31000, Toulouse, France			The MEMENTO Study Group
Karim	Farid			Memory Resource and Research Centre of Paris Nord, AP-HP, Paris, France			The MEMENTO Study Group
Denis	Fédérico			Memory Resource and Research Centre of Lyon, Hospices Civils de Lyon, Hôpital des Charpennes, F-69000, Lyon, France			The MEMENTO Study Group
Olivier	Felician			Memory Resource and Research Centre of Marseille, CHU de Marseille, Hôpital La Timone, F-13000, Marseille, France			The MEMENTO Study Group
Philippe	Fernandez			Memory Resource and Research Centre of Bordeaux, CHU de Bordeaux, Hôpital Pellegrin, F-33000, Bordeaux, France			The MEMENTO Study Group
Pacôme	Fosse			Memory Resource and Research Centre of Angers, CHU d'Angers, F-49000, Angers			The MEMENTO Study Group
Alexandra	Foubert-Samier			Memory Resource and Research Centre of Bordeaux, CHU de Bordeaux, Hôpital Pellegrin, F-33000, Bordeaux, France			The MEMENTO Study Group
Isabelle	Franck			Memory Resource and Research Centre of Strasbourg, Hôpitaux Universitaires de Strasbourg, F-67000, Strasbourg, France			The MEMENTO Study Group
Pauline	Gaffez			Coordinating Centre, Inserm CIC-1401 Clinical Epidemiology, CHU de Bordeaux, F-33000, Bordeaux, France			The MEMENTO Study Group
Monique	Galitzky			Memory Resource and Research Centre of Toulouse, CHU de Toulouse, Hôpital Purpan, F-31000, Toulouse, France			The MEMENTO Study Group
Céline	Gallazzini-Crepin			Memory Resource and Research Centre of Grenoble, CHU de Grenoble Alpes, Grenoble, France			The MEMENTO Study Group
Radka	Gantchev			Memory Resource and Research Centre of Marseille, CHU de Marseille, Hôpital La Timone, F-13000, Marseille, France			The MEMENTO Study Group
Laurence	Garbarg-Chenon			Memory Clinic, Hôpital Avicenne, AP-HP, Hôpitaux Universitaires Paris-Seine-Saint-Denis, F-93009, Bobigny, France			The MEMENTO Study Group
Guillaume	Gautier			Memory Resource and Research Centre of Marseille, CHU de Marseille, Hôpital La Timone, F-13000, Marseille, France			The MEMENTO Study Group
Emmanuel	Gerardin			Memory Resource and Research Centre of Rouen, Neuroradiology Department, Rouen University Hospital, F-76031, Rouen, France			The MEMENTO Study Group
Claire	Gervais			Memory Resource and Research Centre of Nice, CHU de Nice, Institut Claude Pompidou, F-06100, Nice, France			The MEMENTO Study Group
Jean-Claude	Getenet			Memory Resource and Research Centre of Saint-Etienne, CHU de Saint-Etienne, Hôpital Nord, F-42000, Saint-Etienne, France			The MEMENTO Study Group
Nadine	Girard			Memory Resource and Research Centre of Marseille, CHU de Marseille, Hôpital La Timone, F-13000, Marseille, France			The MEMENTO Study Group
Fabienne	Giraud			Memory Resource and Research Centre of Marseille, CHU de Marseille, Hôpital La Timone, F-13000, Marseille, France			The MEMENTO Study Group
Chantal	Girtanner			Memory Resource and Research Centre of Saint-Etienne, CHU de Saint-Etienne, Hôpital de la Charité, F-42000, Saint-Etienne, France			The MEMENTO Study Group
Valérie	Gissot			Memory Resource and Research Centre of Center Region, CHRU de Tours, Hôpital Bretonneau, F-37000, Tours, France			The MEMENTO Study Group

*First name, last name, and suffix (if applicable) are required and will appear in PubMed.

*First Name and Middle In	*Last Name	*Suffix (eg, Jr, III)	Academic D	Institution	Location (city, stat	Role or Contribution,	Group (if more than 1 Group listed in the byline) and/or Subgroup (eg, Steering Committee)
Caroline	Grangeon			Memory Resource and Research Centre of Nice, CHU de Nice, Institut Claude Pompidou, F-06100, Nice, France			The MEMENTO Study Group
Leslie	Grasset			Coordinating Centre, Inserm CIC-1401 Clinical Epidemiology, CHU de Bordeaux, F-33000, Bordeaux, France			The MEMENTO Study Group
Daniel	Grucker			Memory Resource and Research Centre of Strasbourg, Hôpitaux Universitaires de Strasbourg, F-67000, Strasbourg, France			The MEMENTO Study Group
Eric	Guedj			Memory Resource and Research Centre of Marseille, CHU de Marseille, Hôpital La Timone, F-13000, Marseille, France			The MEMENTO Study Group
Claude	Gueriot			Memory Resource and Research Centre of Marseille, CHU de Marseille, Hôpital La Timone, F-13000, Marseille, France			The MEMENTO Study Group
Yves	Guilhermet			Memory Resource and Research Centre of Lyon, Hospices Civils de Lyon, Hôpital des Charpennes, F-69000, Lyon, France			The MEMENTO Study Group
Rémy	Guillevin			Memory Resource and Research Centre of Poitiers, CHU de Poitiers, Hôpital de La Milétrie, F-86000, Poitiers, France			The MEMENTO Study Group
Marie	Odile Habert			Automated Image Acquisition Processing Center – CATI, Functional Imaginf Laboratory – UMR 678 / Pierre et Marie Curie University – 75634 Paris Cede			The MEMENTO Study Group
Sophie	Haffen			Memory Resource and Research Centre of Besançon, CHU de Besançon, Hôpital Jean Minjoz, Hôpital Saint-Jacques, F-25000, Besançon, France			The MEMENTO Study Group
Didier	Hannequin			Memory Resource and Research Centre of Rouen, Neurology Department, Rouen University Hospital, F-76031, Rouen, France			The MEMENTO Study Group
Sandrine	Harston			Memory Resource and Research Centre of Bordeaux, CHU de Bordeaux, Hôpital Xavier Arnozan, F-33000, Bordeaux, France			The MEMENTO Study Group
Anne	Hitzel			Memory Resource and Research Centre of Toulouse, CHU de Toulouse, Hôpital Purpan, F-31000, Toulouse, France			The MEMENTO Study Group
Caroline	Hommet			Memory Resource and Research Centre of Center Region, CHRU de Tours, Hôpital Bretonneau, F-37000, Tours, France			The MEMENTO Study Group
Claude	Hossein-Foucher			Memory Resource and Research Centre of Lille, CHRU de Lille, Hôpital Roger Salengro, F-59000, Lille, France			The MEMENTO Study Group
Fabrice	Hubele			Memory Resource and Research Centre of Strasbourg, Hôpitaux Universitaires de Strasbourg, F-67000, Strasbourg, France			The MEMENTO Study Group
Agnès	Jacquin-Piques			Memory Resource and Research Centre of Dijon, CHU Dijon Bourgogne, Hôpital du Bocage, Hôpital de Champmaillot, F-21000, Dijon, France			The MEMENTO Study Group
Betty	Jean			Memory Resource and Research Centre of Clermont-Ferrand, CHU de Clermont-Ferrand, F-63000, Clermont-Ferrand, France			The MEMENTO Study Group
Delphine	Jean			Coordinating Centre, Inserm CIC-1401 Clinical Epidemiology, CHU de Bordeaux, F-33000, Bordeaux, France			The MEMENTO Study Group
Joanne	Jenn			Memory Resource and Research Centre of Bordeaux, CHU de Bordeaux, Hôpital Xavier Arnozan, F-33000, Bordeaux, France			The MEMENTO Study Group
Laure	Joly			Memory Resource and Research Centre of Nancy, CHU de Nancy, F-54000, Nancy, France			The MEMENTO Study Group
Thérèse	Jonveaux			Memory Resource and Research Centre of Nancy, CHU de Nancy, F-54000, Nancy, France			The MEMENTO Study Group
Adrien	Julian			Memory Resource and Research Centre of Poitiers, CHU de Poitiers, Hôpital de La Milétrie, F-86000, Poitiers, France			The MEMENTO Study Group
Anisse	Karoun			Coordinating Centre, Inserm CIC-1401 Clinical Epidemiology, CHU de Bordeaux, F-33000, Bordeaux, France			The MEMENTO Study Group
Aurélie	Kas			Laboratoire d'Imagerie Biomédicale, Sorbonne Universités, UPMC Univ Paris 06, Inserm U1146, CNRS UMR 7371, France NeuroSpin, I2BM, Commissar			The MEMENTO Study Group
Anna	Kearney-Schwartz			Memory Resource and Research Centre of Nancy, CHU de Nancy, F-54000, Nancy, France			The MEMENTO Study Group
Alice	Keles			Memory Resource and Research Centre of Nancy, CHU de Nancy, F-54000, Nancy, France			The MEMENTO Study Group
Antony	Kelly			Memory Resource and Research Centre of Clermont-Ferrand, Centre de Lutte contre le Cancer, F-63000, Clermont-Ferrand, France			The MEMENTO Study Group
Nathalie	Keromnes			Memory Resource and Research Centre of Brest, CHRU de Brest, F-29000, Brest, France			The MEMENTO Study Group
Lejla	Koric			Memory Resource and Research Centre of Marseille, CHU de Marseille, Hôpital La Timone, F-13000, Marseille, France			The MEMENTO Study Group
Maxime	Locatelli			Automated Image Acquisition Processing Center – CATI, Functional Imaginf Laboratory – UMR 678 / Pierre et Marie Curie University – 75634 Paris Cede			The MEMENTO Study Group
Alexandre	Krainik			Memory Resource and Research Centre of Grenoble, CHU de Grenoble Alpes, Grenoble, France			The MEMENTO Study Group
Stéphane	Kremer			Memory Resource and Research Centre of Strasbourg, Hôpitaux Universitaires de Strasbourg, F-67000, Strasbourg, France			The MEMENTO Study Group
Florian	Labourée			Memory Resource and Research Centre of Paris Broca, AP-HP, Paris, France			The MEMENTO Study Group
Franck	Lacoeuille			Memory Resource and Research Centre of Angers, CHU d'Angers, F-49000, Angers			The MEMENTO Study Group
Emilie	Laheranne			Coordinating Centre, Inserm CIC-1401 Clinical Epidemiology, CHU de Bordeaux, F-33000, Bordeaux, France			The MEMENTO Study Group
Francoise	Lala			Memory Resource and Research Centre of Toulouse, CHU de Toulouse, Hôpital La Grave-Casselardit, F-31000, Toulouse, France			The MEMENTO Study Group
Chantal	Lamy			Memory Resource and Research of Amiens, CHU Amiens Picardie, F-80000, Amiens, France			The MEMENTO Study Group
Jean-Louis	Laplanche			Memory Resource and Research Centre of Paris Nord, AP-HP, Paris, France			The MEMENTO Study Group
Cyrille	Launay			Memory Resource and Research Centre of Angers, CHU d'Angers, F-49000, Angers			The MEMENTO Study Group
Lisa	Le Scouarnec			Coordinating Centre, Inserm CIC-1401 Clinical Epidemiology, CHU de Bordeaux, F-33000, Bordeaux, France			The MEMENTO Study Group
Stéphane	Lehericy			Institute of Memory and Alzheimer's Disease (IM2A), Centre for NeuroImaging Research (CENIR), Brain and Spine Institute (ICM), UMR S 1127, Departn			The MEMENTO Study Group
Sylvain	Lehmann			Memory Resource and Research Centre of Montpellier, CHU de Montpellier, Hôpital Gui de Chauliac, F-34000, Montpellier, France			The MEMENTO Study Group
Hermine	Lenoir			Memory Resource and Research Centre of Paris Broca, AP-HP, Paris, France			The MEMENTO Study Group
Marcel	Levy			Institute of Memory and Alzheimer's Disease (IM2A), Brain and Spine Institute (ICM), UMR S 1127, Department of Neurology, AP-HP, Pitié-Salpêtrière			The MEMENTO Study Group
Stéphanie	Libercier			Memory Resource and Research Centre of Colmar, Hôpitaux Civils de Colmar, F-68000, Colmar, France			The MEMENTO Study Group
Julie	Lidier			Coordinating Centre, Inserm CIC-1401 Clinical Epidemiology, CHU de Bordeaux, F-33000, Bordeaux, France			The MEMENTO Study Group
Marie-Anne	Mackowiak-Cordoliani			Memory Resource and Research Centre of Lille, CHRU de Lille, Hôpital Roger Salengro, F-59000, Lille, France			The MEMENTO Study Group
Eloi	Magnin			Memory Resource and Research Centre of Besançon, CHU de Besançon, Hôpital Jean Minjoz, Hôpital Saint-Jacques, F-25000, Besançon, France			The MEMENTO Study Group
Zaza	Makaroff			Memory Resource and Research Centre of Lyon, Hospices Civils de Lyon, Hôpital des Charpennes, F-69000, Lyon, France			The MEMENTO Study Group
Jean-François	Mangin			Automated Image Acquisition Processing Center – CATI neurospin, , Institute of Biomedical Imaging Atomic Energy Commission, F-91191 Gif sur Yvette			The MEMENTO Study Group
Athina	Marantidou			Memory Clinic, Hôpital Avicenne, AP-HP, Hôpitaux Universitaires Paris-Seine-Saint-Denis, F-93009, Bobigny, France			The MEMENTO Study Group
Isabelle	Marcet			Memory Resource and Research Centre of Bordeaux, CHU de Bordeaux, Hôpital Pellegrin, F-33000, Bordeaux, France			The MEMENTO Study Group
Olivier	Marcy			Coordinating Centre, Inserm CIC-1401 Clinical Epidemiology, CHU de Bordeaux, F-33000, Bordeaux, France			The MEMENTO Study Group
Cécilia	Marelli			Memory Resource and Research Centre of Montpellier, CHU de Montpellier, Hôpital Gui de Chauliac, F-34000, Montpellier, France			The MEMENTO Study Group
Sophie	Marilier			Memory Resource and Research Centre of Dijon, CHU Dijon Bourgogne, Hôpital du Bocage, Hôpital de Champmaillot, F-21000, Dijon, France			The MEMENTO Study Group
Idalie	Martin			Memory Resource and Research Centre of Lyon, Hospices Civils de Lyon, Hôpital des Charpennes, F-69000, Lyon, France			The MEMENTO Study Group
Olivier	Martinaud			Memory Resource and Research Centre of Rouen, Neurology Department, Rouen University Hospital, F-76031, Rouen, France			The MEMENTO Study Group
Catherine	Martin-Hunyadi			Memory Resource and Research Centre of Strasbourg, Hôpitaux Universitaires de Strasbourg, F-67000, Strasbourg, France			The MEMENTO Study Group
Aïcha	Medjoul			Memory Clinic, Hôpital Avicenne, AP-HP, Hôpitaux Universitaires Paris-Seine-Saint-Denis, F-93009, Bobigny, France			The MEMENTO Study Group
Lauralee	Menier			Coordinating Centre, Inserm CIC-1401 Clinical Epidemiology, CHU de Bordeaux, F-33000, Bordeaux, France			The MEMENTO Study Group
Isabelle	Merlet			Memory Resource and Research Centre of Poitiers, CHU de Poitiers, Hôpital de La Milétrie, F-86000, Poitiers, France			The MEMENTO Study Group
Danielle	Mestas			Memory Resource and Research Centre of Clermont-Ferrand, CHU de Clermont-Ferrand, F-63000, Clermont-Ferrand, France			The MEMENTO Study Group
Marc-Etienne	Meyer			Memory Resource and Research of Amiens, CHU Amiens Picardie, F-80000, Amiens, France			The MEMENTO Study Group
Jean-Marc	Michel			Memory Resource and Research Centre of Colmar, Hôpitaux Civils de Colmar, F-68000, Colmar, France			The MEMENTO Study Group
Agnès	Michon			Institute of Memory and Alzheimer's Disease (IM2A), Brain and Spine Institute (ICM), UMR S 1127, Department of Neurology, AP-HP, Pitié-Salpêtrière			The MEMENTO Study Group
Isabelle	Migeon-Duballet			Memory Resource and Research Centre of Poitiers, CHU de Poitiers, Hôpital de La Milétrie, F-86000, Poitiers, France			The MEMENTO Study Group
Sophie	Mohr			Memory Resource and Research Centre of Dijon, CHU Dijon Bourgogne, Hôpital du Bocage, F-21000, Dijon, France			The MEMENTO Study Group
Karl	Mondon			Memory Resource and Research Centre of Center Region, CHRU de Tours, Hôpital Bretonneau, F-37000, Tours, France			The MEMENTO Study Group

*First name, last name, and suffix (if applicable) are required and will appear in PubMed.

*First Name and Middle In	*Last Name	*Suffix (eg, Jr, III)	Academic D	Institution	Location (city, stat	Role or Contribution,	Group (if more than 1 Group listed in the byline) and/or Subgroup (eg, Steering Committee)
Clément	Morgat			Memory Resource and Research Centre of Bordeaux, CHU de Bordeaux, Hôpital Pellegrin, F-33000, Bordeaux, France			The MEMENTO Study Group
Véronique	Moullart			Memory Resource and Research of Amiens, CHU Amiens Picardie, F-80000, Amiens, France			The MEMENTO Study Group
Christian	Moussard			Memory Resource and Research Centre of Besançon, CHU de Besançon, Hôpital Jean Minjoz, Hôpital Saint-Jacques, F-25000, Besançon, France			The MEMENTO Study Group
Aurélie	Mouton			Memory Resource and Research Centre of Nice, CHU de Nice, Institut Claude Pompidou, F-06100, Nice, France			The MEMENTO Study Group
Izzie	Jacques Namer			Memory Resource and Research Centre of Strasbourg, Hôpitaux Universitaires de Strasbourg, F-67000, Strasbourg, France			The MEMENTO Study Group
Georges	Niewiadomski			Memory Resource and Research Centre of Nice, CHU de Nice, Institut Claude Pompidou, F-06100, Nice, France			The MEMENTO Study Group
Guillaume	Nivaggioni			Memory Resource and Research Centre of Nice, CHU de Nice, Institut Claude Pompidou, F-06100, Nice, France			The MEMENTO Study Group
Marie	Noblet			Memory Resource and Research Centre of Strasbourg, Hôpitaux Universitaires de Strasbourg, F-67000, Strasbourg, France			The MEMENTO Study Group
Michel	Nonent			Memory Resource and Research Centre of Brest, CHRU de Brest, F-29000, Brest, France			The MEMENTO Study Group
Fati	Nourhashemi			Memory Resource and Research Centre of Toulouse, CHU de Toulouse, Hôpital La Grave-Casselardit, F-31000, Toulouse, France			The MEMENTO Study Group
Hélène	Oesterle			Memory Resource and Research Centre of Colmar, Hôpitaux Civils de Colmar, F-68000, Colmar, France			The MEMENTO Study Group
Galdric	Orvoen			Memory Resource and Research Centre of Paris Broca, AP-HP, Paris, France			The MEMENTO Study Group
Pierre-Jean	Ousset			Memory Resource and Research Centre of Toulouse, CHU de Toulouse, Hôpital La Grave-Casselardit, F-31000, Toulouse, France			The MEMENTO Study Group
Amandine	Pallardy			Memory Resource and Research Centre of Nantes, CHU de Nantes, F-44000, Nantes, France			The MEMENTO Study Group
Claire	Paquet			Memory Resource and Research Centre of Paris Nord, AP-HP, Paris, France			The MEMENTO Study Group
Pierre-Yves	Pare			Memory Resource and Research Centre of Angers, CHU d’Angers, F-49000, Angers			The MEMENTO Study Group
Anne	Pasco			Memory Resource and Research Centre of Angers, CHU d’Angers, F-49000, Angers			The MEMENTO Study Group
Pierre	Payoux			Memory Resource and Research Centre of Toulouse, CHU de Toulouse, Hôpital Purpan, F-31000, Toulouse, France			The MEMENTO Study Group
Cécile	Pays			Memory Resource and Research Centre of Montpellier, CHU de Montpellier, Hôpital Gui de Chauliac, F-34000, Montpellier, France			The MEMENTO Study Group
Isabelle	Pellegrin			Biological Research Centre, CHU de Bordeaux, F-33000, Bordeaux, France			The MEMENTO Study Group
Rémy	Perdrisot			Memory Resource and Research Centre of Poitiers, CHU de Poitiers, Hôpital de La Milétrie, F-86000, Poitiers, France			The MEMENTO Study Group
Bertille	Perin			Memory Resource and Research of Amiens, CHU Amiens Picardie, F-80000, Amiens, France			The MEMENTO Study Group
Christine	Perret-Guillaume			Memory Resource and Research Centre of Nancy, CHU de Nancy, F-54000, Nancy, France			The MEMENTO Study Group
Sophie	Perusat			Coordinating Centre, Inserm CIC-1401 Clinical Epidemiology, CHU de Bordeaux, F-33000, Bordeaux, France			The MEMENTO Study Group
Grégory	Petyt			Memory Resource and Research Centre of Lille, CHRU de Lille, Hôpital Roger Salengro, F-59000, Lille, France			The MEMENTO Study Group
Nathalie	Philippi			Memory Resource and Research Centre of Strasbourg, Hôpitaux Universitaires de Strasbourg, F-67000, Strasbourg, France			The MEMENTO Study Group
Geneviève	Pinganaud			Memory Resource and Research Centre of Bordeaux, CHU de Bordeaux, Hôpital Xavier Arnozan, F-33000, Bordeaux, France			The MEMENTO Study Group
Vincent	Planche			Memory Resource and Research Centre of Bordeaux, CHU de Bordeaux, Hôpital Pellegrin, F-33000, Bordeaux, France			The MEMENTO Study Group
Matthieu	Plichart			Memory Resource and Research Centre of Paris Broca, AP-HP, Paris, France			The MEMENTO Study Group
Gabriel	Pop			Memory Clinic, Hôpital Avicenne, AP-HP, Hôpitaux Universitaires Paris-Seine-Saint-Denis, F-93009, Bobigny, France			The MEMENTO Study Group
Michèle	Puel			Memory Resource and Research Centre of Toulouse, CHU de Toulouse, Hôpital Purpan, F-31000, Toulouse, France			The MEMENTO Study Group
Mathieu	Queneau			Memory Resource and Research Centre of Paris Nord, Centre Cardiologique du Nord, Paris, France			The MEMENTO Study Group
Solène	Querellou			Memory Resource and Research Centre of Brest, CHRU de Brest, F-29000, Brest, France			The MEMENTO Study Group
Muriel	Quillard-Muraine			Memory Resource and Research Centre of Rouen, Neurology Department, Rouen University Hospital, F-76031, Rouen, France			The MEMENTO Study Group
Valérie	Quipourt			Memory Resource and Research Centre of Dijon, CHU Dijon Bourgogne, Hôpital du Bocage, Hôpital de Champmaillot, F-21000, Dijon, France			The MEMENTO Study Group
Chloé	Rachez			Memory Resource and Research Centre of Clermont-Ferrand, CHU de Clermont-Ferrand, F-63000, Clermont-Ferrand, France			The MEMENTO Study Group
Micheline	Razzouk-Cadet			Memory Resource and Research Centre of Nice, CHU de Nice, Institut Claude Pompidou, F-06100, Nice, France			The MEMENTO Study Group
Anne-Sophie	Rigaud			Memory Resource and Research Centre of Paris Broca, AP-HP, Paris, France			The MEMENTO Study Group
Hélène	Robin-Ismer			Memory Resource and Research Centre of Strasbourg, Hôpitaux Universitaires de Strasbourg, F-67000, Strasbourg, France			The MEMENTO Study Group
Mathieu	Rodallec			Memory Resource and Research Centre of Paris Nord, Centre Cardiologique du Nord, Paris, France			The MEMENTO Study Group
Yves	Rolland			Memory Resource and Research Centre of Toulouse, CHU de Toulouse, Hôpital La Grave-Casselardit, F-31000, Toulouse, France			The MEMENTO Study Group
Adeline	Rollin-Sillaire			Memory Resource and Research Centre of Lille, CHRU de Lille, Hôpital Roger Salengro, F-59000, Lille, France			The MEMENTO Study Group
Stéphanie	Roseng			Coordinating Centre, Inserm CIC-1401 Clinical Epidemiology, CHU de Bordeaux, F-33000, Bordeaux, France			The MEMENTO Study Group
Olivier	Rouaud			Memory Resource and Research Centre of Dijon, CHU Dijon Bourgogne, Hôpital du Bocage, Hôpital de Champmaillot, F-21000, Dijon, France			The MEMENTO Study Group
Caroline	Roubaud			Memory Resource and Research Centre of Lyon, Hospices Civils de Lyon, Hôpital des Charpennes, F-69000, Lyon, France			The MEMENTO Study Group
Isabelle	Rouch			Memory Resource and Research Centre of Lyon, Hospices Civils de Lyon, Hôpital des Charpennes, F-69000, Lyon, France			The MEMENTO Study Group
Julie	Roux			Memory Resource and Research Centre of Grenoble, CHU de Grenoble Alpes, Grenoble, France			The MEMENTO Study Group
Guillaume	Sacco			Memory Resource and Research Centre of Nice, CHU de Nice, Institut Claude Pompidou, F-06100, Nice, France			The MEMENTO Study Group
Pierre-Yves	Salaun			Memory Resource and Research Centre of Brest, CHRU de Brest, F-29000, Brest, France			The MEMENTO Study Group
François	Salmon			Memory Resource and Research Centre of Poitiers, CHU de Poitiers, Hôpital de La Milétrie, F-86000, Poitiers, France			The MEMENTO Study Group
Alicia	Sanchez			Memory Resource and Research Centre of Saint-Etienne, CHU de Saint-Etienne, Hôpital Nord, F-42000, Saint-Etienne, France			The MEMENTO Study Group
Maria-Joao	Santiago-Ribeiro			Memory Resource and Research Centre of Center Region, CHRU de Tours, Hôpital Bretonneau, F-37000, Tours, France			The MEMENTO Study Group
Alain	Sarciron			Memory Resource and Research Centre of Lyon, Hospices Civils de Lyon, Hôpital des Charpennes, F-69000, Lyon, France			The MEMENTO Study Group
Nathalie	Sastre-Hengan			Memory Resource and Research Centre of Toulouse, CHU de Toulouse, Hôpital La Grave-Casselardit, F-31000, Toulouse, France			The MEMENTO Study Group
Mathilde	Sauvée			Memory Resource and Research Centre of Grenoble, CHU de Grenoble Alpes, Grenoble, France			The MEMENTO Study Group
Helen	Savarieau			Coordinating Centre, Inserm CIC-1401 Clinical Epidemiology, CHU de Bordeaux, F-33000, Bordeaux, France			The MEMENTO Study Group
Christian	Scheiber			Memory Resource and Research Centre of Lyon, Hospices Civils de Lyon, Hôpital des Charpennes, F-69000, Lyon, France			The MEMENTO Study Group
Anne-Marie	Schneider			Memory Resource and Research Centre of Strasbourg, Hôpitaux Universitaires de Strasbourg, F-67000, Strasbourg, France			The MEMENTO Study Group
Franck	Semah			Memory Resource and Research Centre of Lille, CHRU de Lille, Hôpital Roger Salengro, F-59000, Lille, France			The MEMENTO Study Group
Amélie	Serra			Memory Resource and Research Centre of Grenoble, CHU de Grenoble Alpes, Grenoble, France			The MEMENTO Study Group
Marie-Laure	Seux			Memory Resource and Research Centre of Paris Broca, AP-HP, Paris, France			The MEMENTO Study Group
Hélène	Sordet-Guépet			Memory Resource and Research Centre of Dijon, CHU Dijon Bourgogne, Hôpital du Bocage, Hôpital de Champmaillot, F-21000, Dijon, France			The MEMENTO Study Group
Maria	Eugenia Soto			Memory Resource and Research Centre of Toulouse, CHU de Toulouse, Hôpital La Grave-Casselardit, F-31000, Toulouse, France			The MEMENTO Study Group
Sullivan	Marie			Automated Image Acquisition Processing Center – CATI, Functional Imaginf Laboratory – UMR 678 / Pierre et Marie Curie University – 75634 Paris Cede			The MEMENTO Study Group
Mathieu	Tafani			Memory Resource and Research Centre of Toulouse, CHU de Toulouse, Hôpital Purpan, F-31000, Toulouse, France			The MEMENTO Study Group
Jean-Yves	Tanguy			Memory Resource and Research Centre of Angers, CHU d’Angers, F-49000, Angers			The MEMENTO Study Group
Michael	Taroux			Memory Resource and Research Centre of Dijon, CHU Dijon Bourgogne, Hôpital du Bocage, Hôpital de Champmaillot, F-21000, Dijon, France			The MEMENTO Study Group
Marc	Teichmann			Institute of Memory and Alzheimer's Disease (IM2A), Brain and Spine Institute (ICM), UMR S 1127, Department of Neurology, AP-HP, Pitié-Salpêtrière			The MEMENTO Study Group

Supplemental Online Content: Nonauthor Collaborators
*First name, last name, and suffix (if applicable) are required and will appear in PubMed.

*First Name and Middle In	*Last Name	*Suffix (eg, Jr, III)	Academic D	Institution	Location (city, stat	Role or Contribution,	Group (if more than 1 Group listed in the byline) and/or Subgroup (eg, Steering Committee)
Catherine	Terrat			Memory Resource and Research Centre of Saint-Etienne, CHU de Saint-Etienne, Hôpital de la Charité, F-42000, Saint-Etienne, France			The MEMENTO Study Group
Jamila	Thabet			Memory Clinic, Hôpital Avicenne, AP-HP, Hôpitaux Universitaires Paris-Seine-Saint-Denis, F-93009, Bobigny, France			The MEMENTO Study Group
Claire	Thalamas			Memory Resource and Research Centre of Toulouse, CHU de Toulouse, Hôpital Purpan, F-31000, Toulouse, France			The MEMENTO Study Group
Nathalie	Thierry			Coordinating Centre, Inserm CIC-1401 Clinical Epidemiology, CHU de Bordeaux, F-33000, Bordeaux, France			The MEMENTO Study Group
Catherine	Thomas-Anterion			Memory Resource and Research Centre of Saint-Etienne, CHU de Saint-Etienne, Hôpital Nord, F-42000, Saint-Etienne, France			The MEMENTO Study Group
Anne-Cécile	Troussière			Memory Resource and Research Centre of Lille, CHRU de Lille, Hôpital Roger Salengro, F-59000, Lille, France			The MEMENTO Study Group
Renata	Ursu			Memory Clinic, Hôpital Avicenne, AP-HP, Hôpitaux Universitaires Paris-Seine-Saint-Denis, F-93009, Bobigny, France			The MEMENTO Study Group
Pierre	Vera			Memory Resource and Research Centre of Rouen, CLCC Henri Becquerel, Rouen, France			The MEMENTO Study Group
Martine	Vercelletto			Memory Resource and Research Centre of Nantes, CHU de Nantes, F-44000, Nantes, France			The MEMENTO Study Group
Olivier	Vercruysse			Memory Resource and Research Centre of Lille, CHRU de Lille, Hôpital Roger Salengro, F-59000, Lille, France			The MEMENTO Study Group
Antoine	Verger			Memory Resource and Research Centre of Nancy, CHU de Nancy, F-54000, Nancy, France			The MEMENTO Study Group
Philippe	Viau			Memory Resource and Research Centre of Nice, CHU de Nice, Institut Claude Pompidou, F-06100, Nice, France			The MEMENTO Study Group
Marie-Neige	Videau			Memory Resource and Research Centre of Bordeaux, CHU de Bordeaux, Hôpital Xavier Arnozan, F-33000, Bordeaux, France			The MEMENTO Study Group
Thierry	Voisin			Memory Resource and Research Centre of Toulouse, CHU de Toulouse, Hôpital La Grave-Casselardit, F-31000, Toulouse, France			The MEMENTO Study Group
Nathalie	Wagemann			Memory Resource and Research Centre of Nantes, CHU de Nantes, F-44000, Nantes, France			The MEMENTO Study Group
Aziza	Waissi-Sediq			Memory Resource and Research Centre of Lyon, Hospices Civils de Lyon, Hôpital des Charpennes, F-69000, Lyon, France			The MEMENTO Study Group
Jing	Xie			Memory Resource and Research Centre of Lyon, Hospices Civils de Lyon, Hôpital des Charpennes, F-69000, Lyon, France			The MEMENTO Study Group
Nathanaëlle	Yeni			Laboratoire d'Imagerie Biomédicale, Sorbonne Universités, UPMC Univ Paris 06, Inserm U1146, CNRS UMR 7371, France NeuroSpin, I2BM, Commissar			The MEMENTO Study Group
Michel	Zanca			Memory Resource and Research Centre of Montpellier, CHU de Montpellier, Hôpital Gui de Chauliac, F-34000, Montpellier, France			The MEMENTO Study Group
Jean	Zinszner			Memory Clinic, Hôpital Avicenne, AP-HP, Hôpitaux Universitaires Paris-Seine-Saint-Denis, F-93009, Bobigny, France			The MEMENTO Study Group
Olivier	Hanon			Université de Paris, EA 4468, APHP, Hopital Broca, Memory Resource and Research Centre of de Paris-Broca-Ile de France, F-75013 Paris, France			The BALTAZAR Study Group
Frédéric	Blanc			Université de Strasbourg, Hôpitaux Universitaires de Strasbourg, CM2R, pôle de Gériatrie, Laboratoire ICube, FMTS, CNRS, équipe IMIS, F-67000 Strasb			The BALTAZAR Study Group
Yasmina	Boudali			Université de Paris, EA 4468, APHP, Hopital Broca, Memory Resource and Research Centre of de Paris-Broca-Ile de France, F-75013 Paris, France			The BALTAZAR Study Group
Audrey	Gabelle			Université de Montpellier, CHU Montpellier, Memory Research and Resources center of Montpellier, department of Neurology, Inserm INM NeuroPEP			The BALTAZAR Study Group
Jacques	Touchon			Université de Montpellier, CHU Montpellier, Memory Research and Resources center of Montpellier, department of Neurology, Inserm INM NeuroPEP			The BALTAZAR Study Group
Marie-Laure	Seux			Université de Paris, EA 4468, APHP, Hopital Broca, Memory Resource and Research Centre of de Paris-Broca-Ile de France, F-75013 Paris, France			The BALTAZAR Study Group
Hermine	Lenoir			Université de Paris, EA 4468, APHP, Hopital Broca, Memory Resource and Research Centre of de Paris-Broca-Ile de France, F-75013 Paris, France			The BALTAZAR Study Group
Catherine	Bayle			Université de Paris, EA 4468, APHP, Hopital Broca, Memory Resource and Research Centre of de Paris-Broca-Ile de France, F-75013 Paris, France			The BALTAZAR Study Group
Stéphanie	Bombois			Univ. Lille, Inserm, CHU Lille, U1172-LilNCog, LiCEND, LabEx DISTALZ, F-59000 Lille, France.			The BALTAZAR Study Group
Christine	Delmaire			Univ. Lille, Inserm, CHU Lille, U1172-LilNCog, LiCEND, LabEx DISTALZ, F-59000 Lille, France.			The BALTAZAR Study Group
Xavier	Delbeuck			Univ. Lille, Inserm U1171 Degenerative and Vascular Cognitive Disorders, F-59000 Lille, France.			The BALTAZAR Study Group
Florence	Moulin			Université de Paris, EA 4468, APHP, Hopital Broca, Memory Resource and Research Centre of de Paris-Broca-Ile de France, F-75013 Paris, France			The BALTAZAR Study Group
Emmanuelle	Duron			Université Paris-Saclay, APHP, Hôpital Paul Brousse, département de gériatrie, Équipe MOODS, Inserm 1178, F-94800 Villejuif, France			The BALTAZAR Study Group
Florence	Latour			Centre Hospitalier de la Côte Basque, Department of Gerontology, F-64100 Bayonne, France			The BALTAZAR Study Group
Matthieu	Plichart			Université de Paris, EA 4468, APHP, Hopital Broca, Memory Resource and Research Centre of de Paris-Broca-Ile de France, F-75013 Paris, France			The BALTAZAR Study Group
Sophie	Pichierri			Université de Nantes, EA 4334 Movement-Interactions-Performance, CHU Nantes, Memory Research Resource Center of Nantes, Department of clinica			The BALTAZAR Study Group
Galdric	Orvoën			Université de Paris, EA 4468, APHP, Hopital Broca, Memory Resource and Research Centre of de Paris-Broca-Ile de France, F-75013 Paris, France			The BALTAZAR Study Group
Evelyne	Galbrun			Sorbonne Université, APHP, Centre Hospitalier Dupuytren, Department of Gérontology 2, F-91210 Draveil, France			The BALTAZAR Study Group
Giovanni	Castelnovo			CHU de Nimes, Hôpital Caremeau, Neurology Department, F-30029 Nimes, France.			The BALTAZAR Study Group
Lisette	Volpe-Gillot			Hôpital Léopold Bellan, Service de Neuro-Psycho-Gériatrie, Memory Clinic, F-75014 Paris, France.			The BALTAZAR Study Group
Florien	Labourée			Université de Paris, EA 4468, APHP, Hopital Broca, Memory Resource and Research Centre of de Paris-Broca-Ile de France, F-75013 Paris, France			The BALTAZAR Study Group
Pascaline	Cassagnaud			Univ. Lille, CHU de Lille, Memory Resource and Research Centre of Lille, Department of Neurology, F-59000 Lille, France.			The BALTAZAR Study Group
Claire	Paquet			Université de Paris, APHP, Groupe Hospitalier Saint Louis-LariboisièreFernand Widal, Center of Cognitive Neurology, F-75010 Paris, France.			The BALTAZAR Study Group
Françoise	Lala			Université de Toulouse III, CHU La Grave-Casselardit, Memory Resource and Research Centre of Midi-Pyrénées, F-31300 Toulouse, France			The BALTAZAR Study Group
Bruno	Vellas			Université de Toulouse III, CHU La Grave-Casselardit, Memory Resource and Research Centre of Midi-Pyrénées, F-31300 Toulouse, France			The BALTAZAR Study Group
Julien	Dumurgier			Université de Paris, APHP, Groupe Hospitalier Saint Louis-LariboisièreFernand Widal, Center of Cognitive Neurology, F-75010 Paris, France.			The BALTAZAR Study Group
Anne-Sophie	Rigaud			Université de Paris, EA 4468, APHP, Hopital Broca, Memory Resource and Research Centre of de Paris-Broca-Ile de France, F-75013 Paris, France			The BALTAZAR Study Group
Christine	Perret-Guillaume			Université de Lorraine, CHRU de Nancy, Memory Resource and Research Centre of Lorraine, F-54500 Vandoeuvre-lès-Nancy, France			The BALTAZAR Study Group
Eliana	Alonso			Université de Paris, APHP, Hôpital europeen Georges Pompidou, Service de Gériatrie, F-75015, Paris, France.			The BALTAZAR Study Group
Foucaud	du Boisgueheneuc			CHU de Poitiers, Memory Resource and Research Centre of Poitiers, F-86000 Poitiers, France.			The BALTAZAR Study Group
Laurence	Hugonot-Diener			Université de Paris, EA 4468, APHP, Hopital Broca, Memory Resource and Research Centre of de Paris-Broca-Ile de France, F-75013 Paris, France			The BALTAZAR Study Group
Adeline	Rollin-Sillaire			Univ. Lille, CHU de Lille, Memory Resource and Research Centre of Lille, Department of Neurology, F-59000 Lille, France.			The BALTAZAR Study Group
Olivier	Martinaud			CHU Charles Nicolle, Memory Resource and Research Centre of HauteNormandie, F-76000 Rouen, France.			The BALTAZAR Study Group
Clémence	Boully			Université de Paris, EA 4468, APHP, Hopital Broca, Memory Resource and Research Centre of de Paris-Broca-Ile de France, F-75013 Paris, France			The BALTAZAR Study Group
Yann	Spivac			APHP, Centre Hospitalier Émile-Roux, Department of Gérontology 1, F-94450 Limeil-Brévannes, France.			The BALTAZAR Study Group
Agnès	Devendeville			CHU d'Amiens-Picardie, Memory Resource and Research Centre of AmiensPicardie, F-80000 Amiens, France.			The BALTAZAR Study Group
Joël	Belmin			Sorbonne Université, APHP, Hôpitaux Universitaires Pitie-Salpêtrière-Charles Foix, Service de Gériatrie Ambulatoire, F-75013 Paris, France.			The BALTAZAR Study Group
Philippe	Robert			Université Côte d'Azur, CHU de Nice, Memory Research Resource Center of Nice, CoBTek lab, F-06100 Nice, France			The BALTAZAR Study Group
Thierry	Dantoine			CHU de Limoges, Memory Research Resource Center of Limoges, F-87000 Limoges, France.			The BALTAZAR Study Group
Laure	Caillard			Université de Paris, EA 4468, APHP, Hopital Broca, Memory Resource and Research Centre of de Paris-Broca-Ile de France, F-75013 Paris, France			The BALTAZAR Study Group
David	Wallon			Normandie Univ, UNIROUEN, Inserm U1245, CHU de Rouen, Department of Neurology and CNR-MAJ, Normandy Center for Genomic and Personalized			The BALTAZAR Study Group
Didier	Hannequin			CHU Charles Nicolle, Memory Resource and Research Centre of HauteNormandie, F-76000 Rouen, France.			The BALTAZAR Study Group
Nathalie	Sastre			Université de Toulouse III, CHU La Grave-Casselardit, Memory Resource and Research Centre of Midi-Pyrénées, F-31300 Toulouse, France			The BALTAZAR Study Group
Sophie	Haffen			CHU de Besançon, Memory Resource and Research Centre of BesançonFranche-Comté, F-25000 Besançon, France			The BALTAZAR Study Group
Anna	Kearney-Schwartz			Université de Lorraine, CHRU de Nancy, Memory Resource and Research Centre of Lorraine, F-54500 Vandoeuvre-lès-Nancy, France			The BALTAZAR Study Group
Jean-Luc	Novella			Université de Reims Champagne-Ardenne, EA 3797, CHU de Reims, Memory Resource and Research Centre of Champagne-Ardenne, F-51100 Reims, Fr			The BALTAZAR Study Group
Vincent	Deramecourt			Univ. Lille, CHU de Lille, Memory Resource and Research Centre of Lille, Department of Neurology, F-59000 Lille, France.			The BALTAZAR Study Group
Valérie	Chauvire			CHU d'Angers, Memory Resource and Research Centre of Angers, F-49000 Angers, France.			The BALTAZAR Study Group

*First Name and Middle In	*Last Name	*Suffix (eg, Jr, III)	Academic D	Institution	Location (city, stat	Role or Contribution,	Group (if more than 1 Group listed in the byline) and/or Subgroup (eg, Steering Committee)
Gabiel	Abitbol			Université de Paris, EA 4468, APHP, Hopital Broca, Memory Resource and Research Centre of de Paris-Broca-Ile de France, F-75013 Paris, France			The BALTAZAR Study Group
Nathalie	Schwald			APHP, Centre Hospitalier Émile-Roux, Department of Gérontology 1, F-94450 Limeil-Brévannes, France.			The BALTAZAR Study Group
Caroline	Hommet			CHRU de Tours, Memory Resource and Research Centre of Tours, F-37000 Tours, France			The BALTAZAR Study Group
François	Sellal			Université de Strasbourg, CHRU de Strasbourg, Memory Resource and Research Centre of Strasbourg/Colmar, Inserm U-118, F-67000 Strasbourg, Fran			The BALTAZAR Study Group
Marie-Ange	Cariot			Université de Paris, APHP, Hôpital europeen Georges Pompidou, Service de Gériatrie, F-75015, Paris, France.			The BALTAZAR Study Group
Mohamed	Abdellaoui			Univ Paris Est Creteil, EA 4391 Excitabilité Nerveuse et Thérapeutique, CHU Henri Mondor, Department of Neurology, F-94000 Créteil, France			The BALTAZAR Study Group
Sarah	Benisty			Hôpital Fondation Rothschild, Department of Neurology, F-75019 Paris, France			The BALTAZAR Study Group
Salim	Gherabli			Université de Paris, EA 4468, APHP, Hopital Broca, Memory Resource and Research Centre of de Paris-Broca-Ile de France, F-75013 Paris, France			The BALTAZAR Study Group
Pierre	Anthony			Université de Strasbourg, CHRU de Strasbourg, Memory Resource and Research Centre of Strasbourg/Colmar, Inserm U-118, F-67000 Strasbourg, Fran			The BALTAZAR Study Group
Frédéric	Bloch			CHU d'Amiens-Picardie, Department of Gerontology, F-80000 Amiens, France.			The BALTAZAR Study Group
Nathalie	Charasz			Université de Paris, EA 4468, APHP, Hopital Broca, Memory Resource and Research Centre of de Paris-Broca-Ile de France, F-75013 Paris, France			The BALTAZAR Study Group
Sophie	Chauvelier			Université de Paris, EA 4468, APHP, Hopital Broca, Memory Resource and Research Centre of de Paris-Broca-Ile de France, F-75013 Paris, France			The BALTAZAR Study Group
Jean-Yves	Gaubert			Université de Paris, EA 4468, APHP, Hopital Broca, Memory Resource and Research Centre of de Paris-Broca-Ile de France, F-75013 Paris, France			The BALTAZAR Study Group
Guillaume	Sacco			Université Côte d'Azur, CHU de Nice, Memory Research Resource Center of Nice, CoBTek lab, F-06100 Nice, France			The BALTAZAR Study Group
Olivier	Guerin			Université Côte d'Azur, CHU de Nice, Memory Research Resource Center of Nice, CoBTek lab, F-06100 Nice, France			The BALTAZAR Study Group
Jacques	Boddaert			Sorbonne Université, APHP, Hôpitaux Universitaires Pitie-Salpêtrière-Charles Foix, Memory Resource and Research Centre, Centre des Maladies Cognit			The BALTAZAR Study Group
Marc	Paccalin			CHU de Poitiers, Memory Resource and Research Centre of Poitiers, F-86000 Poitiers, France.			The BALTAZAR Study Group
Marie-Anne	Mackowiak			Univ. Lille, CHU de Lille, Memory Resource and Research Centre of Lille, Department of Neurology, F-59000 Lille, France.			The BALTAZAR Study Group
Marie-Thérèse	Rabus			Sorbonne Université, APHP, Centre Hospitalier Dupuytren, Department of Gérontology 2, F-91210 Draveil, France			The BALTAZAR Study Group
Valérie	Gissot			Université François-Rabelais de Tours, CHRU de Tours, Memory Resource and Research Centre of Tours, Inserm CIC 1415, F-37000 Tours, France			The BALTAZAR Study Group
Athanase	Benetos			Université de Lorraine, CHRU de Nancy, Memory Resource and Research Centre of Lorraine, F-54500 Vandoeuvre-lès-Nancy, France			The BALTAZAR Study Group
Candice	Picard			CHU d'Amiens-Picardie, Memory Resource and Research Centre of AmiensPicardie, F-80000 Amiens, France.			The BALTAZAR Study Group
Céline	Guillemaud			Sorbonne Université, APHP, Hôpitaux Universitaires Pitie-Salpêtrière-Charles Foix, Memory Resource and Research Centre, Centre des Maladies Cognit			The BALTAZAR Study Group
Gilles	Berrut			Université de Nantes, EA 4334 Movement-Interactions-Performance, CHU Nantes, Memory Research Resource Center of Nantes, Department of clinica			The BALTAZAR Study Group
Claire	Gervais			Université Côte d'Azur, CHU de Nice, Memory Research Resource Center of Nice, CoBTek lab, F-06100 Nice, France			The BALTAZAR Study Group
Jaques	Hugon			Université de Paris, APHP, Groupe Hospitalier Saint Louis-LariboisièreFernand Widal, Center of Cognitive Neurology, F-75010 Paris, France.			The BALTAZAR Study Group
Jean-Marc	Michel			Université de Strasbourg, CHRU de Strasbourg, Memory Resource and Research Centre of Strasbourg/Colmar, Inserm U-118, F-67000 Strasbourg, Fran			The BALTAZAR Study Group
JeanPhilippe	David			APHP, Centre Hospitalier Émile-Roux, Department of Gérontology 1, F-94450 Limeil-Brévannes, France.			The BALTAZAR Study Group
Marion	Paulin			Univ. Lille, CHU de Lille, Memory Resource and Research Centre of Lille, Department of Neurology, F-59000 Lille, France.			The BALTAZAR Study Group
Pierre-Jean	Ousset			Université de Toulouse III, CHU La Grave-Casselardit, Memory Resource and Research Centre of Midi-Pyrénées, F-31300 Toulouse, France			The BALTAZAR Study Group
Pierre	Vandel			Université Bourgogne Franche-Comté, Laboratoire de Recherches Inté- gratives en Neurosciences et Psychologie Cognitive, CHU de Besançon, Memory			The BALTAZAR Study Group
Sylvie	Pariel			Sorbonne Université, APHP, Hôpitaux Universitaires Pitie-Salpêtrière-Charles Foix, Service de Gériatrie Ambulatoire, F-75013 Paris, France.			The BALTAZAR Study Group
Vincent	Camus			Université François-Rabelais de Tours, CHRU de Tours, UMR Inserm U1253, F-37000 Tours, France			The BALTAZAR Study Group
Anne	Chawakilian			Université de Paris, EA 4468, APHP, Hopital Broca, Memory Resource and Research Centre of de Paris-Broca-Ile de France, F-75013 Paris, France			The BALTAZAR Study Group
Léna	Kermanac'h			Université de Paris, EA 4468, APHP, Hopital Broca, Memory Resource and Research Centre of de Paris-Broca-Ile de France, F-75013 Paris, France			The BALTAZAR Study Group
Anne-Cécile	Troussiere			Univ. Lille, CHU de Lille, Memory Resource and Research Centre of Lille, Department of Neurology, F-59000 Lille, France.			The BALTAZAR Study Group
Cécile	Adam			CHU de Limoges, Memory Research Resource Center of Limoges, F-87000 Limoges, France.			The BALTAZAR Study Group
Diane	Dupuy			CHU d'Amiens-Picardie, Memory Resource and Research Centre of AmiensPicardie, F-80000 Amiens, France.			The BALTAZAR Study Group
Elena	Paillaud			Université de Paris, APHP, Hôpital europeen Georges Pompidou, Service de Gériatrie, F-75015, Paris, France.			The BALTAZAR Study Group
Hélène	Briault			Sorbonne Université, APHP, Centre Hospitalier Dupuytren, Department of Gérontology 2, F-91210 Draveil, France			The BALTAZAR Study Group
Isabelle	Saulnier			Université de Limoges, EA 6310 HAVAE, CHU de Limoges, Memory Research Resource Center of Limoges, F-87000 Limoges, France			The BALTAZAR Study Group
Karl	Mondon			Université François-Rabelais de Tours, CHRU de Tours, UMR Inserm U1253, F-37000 Tours, France			The BALTAZAR Study Group
Marie-Agnès	Picat			CHU de Limoges, Memory Research Resource Center of Limoges, F-87000 Limoges, France.			The BALTAZAR Study Group
Marie	Laurent			Université de Paris, APHP, Hôpital europeen Georges Pompidou, Service de Gériatrie, F-75015, Paris, France.			The BALTAZAR Study Group
Olivier	Godefroy			CHU d'Amiens-Picardie, Memory Resource and Research Centre of AmiensPicardie, F-80000 Amiens, France.			The BALTAZAR Study Group
Rezki	Daheb			Université de Paris, APHP, Hôpital europeen Georges Pompidou, Service de Gériatrie, F-75015, Paris, France.			The BALTAZAR Study Group
Stéphanie	Libercier			Université de Strasbourg, CHRU de Strasbourg, Memory Resource and Research Centre of Strasbourg/Colmar, Inserm U-118, F-67000 Strasbourg, Fran			The BALTAZAR Study Group
Djamila	Krabchi			Université de Paris, EA 4468, APHP, Hopital Broca, Memory Resource and Research Centre of de Paris-Broca-Ile de France, F-75013 Paris, France			The BALTAZAR Study Group
Marie	Chupin			Université Paris-Saclay, Neurospin, CEA, CNRS, cati-neuroimaging.com, CATI Multicenter Neuroimaging Platform, F-91190 Gif-sur-Yvette, France.			The BALTAZAR Study Group
Jean-Sébastien	Vidal			Université de Paris, EA 4468, APHP, Hopital Broca, Memory Resource and Research Centre of de Paris-Broca-Ile de France, F-75013 Paris, France			The BALTAZAR Study Group
Edouard	Chaussade			Université de Paris, EA 4468, APHP, Hopital Broca, Memory Resource and Research Centre of de Paris-Broca-Ile de France, F-75013 Paris, France			The BALTAZAR Study Group
Christiane	Baret-Rose			Université de Paris, Institute of Psychiatric and Neurosciences, Inserm UMR-S 1266, F-75014 Paris, France			The BALTAZAR Study Group
Sylvain	Lehmann			Université de Montpellier, CHU Montpellier, LBPC, Inserm, F-34000 Montpellier, France.			The BALTAZAR Study Group
Bernadette	Allinquant			Université de Paris, Institute of Psychiatric and Neurosciences, Inserm UMR-S 1266, F-75014 Paris, France			The BALTAZAR Study Group
Susanna	Schraen-Maschke			Univ. Lille, Inserm, CHU Lille, U1172-LilNCog, LiCEND, LabEx DISTALZ, F-59000 Lille, France.			The BALTAZAR Study Group
