# Supplementary material for: Cognitive Phenotyping and Interpretation of Alzheimer Blood Biomarkers
Source: JAMA Neurol. 2025 Apr 4;82(5):506–15. doi: 10.1001/jamaneurol.2025.0142 (PMC11971688; doi:10.1001/jamaneurol.2025.0142)
Supplement: Supplement 3. — Data Sharing Statement. [file jamaneurol-e250142-s003.pdf]

Data Sharing Statement

Bouteloup. Cognitive Phenotyping and Interpretation of Alzheimer Blood Biomarkers. *JAMA Neurol*. Published April 04, 2025. doi:10.1001/jamaneurol.2025.0142

Data
Data available: Yes
Data types: Other (please specify)
Additional Information: Analyzed data could be available through the DPUK platform.
How to access data: https://www.dementiasplatform.uk/
When available: With publication

Supporting Documents
Document types: Other (please specify)
Additional Information: Database documentation
How to access documents: https://www.dementiasplatform.uk/
When available: With publication

Additional Information
Who can access the data: Researchers whose proposed use of the data has been approved
Types of analyses: For any purpose
Mechanisms of data availability: After approval of a proposal
